# Supplementary material for: Immunization strategies in networks with missing data
Source: PLoS Comput Biol. 2020 Jul 9;16(7):e1007897. doi: 10.1371/journal.pcbi.1007897 (PMC7386582; doi:10.1371/journal.pcbi.1007897)
Supplement: S1 Appendix — Additional analyses and figures to support the results of the paper. (PDF) [file pcbi.1007897.s001.pdf]

Supporting Information for: "Immunization Strategies in Networks with Missing Data"

Samuel F. Rosenblatt*[1,2], Jeffrey A. Smith[3], G. Robin Gauthier[3], Laurent Hébert-Dufresne[1,2]

1 Department of Computer Science, University of Vermont, Burlington, VT 05405
2 Vermont Complex Systems Center, University of Vermont, Burlington, VT 05405
3 Department of Sociology, University of Nebraska-Lincoln, Lincoln, NE 68588

* Samuel.F.Rosenblatt@uvm.edu

1 Results on Mechanistically Generated Networks

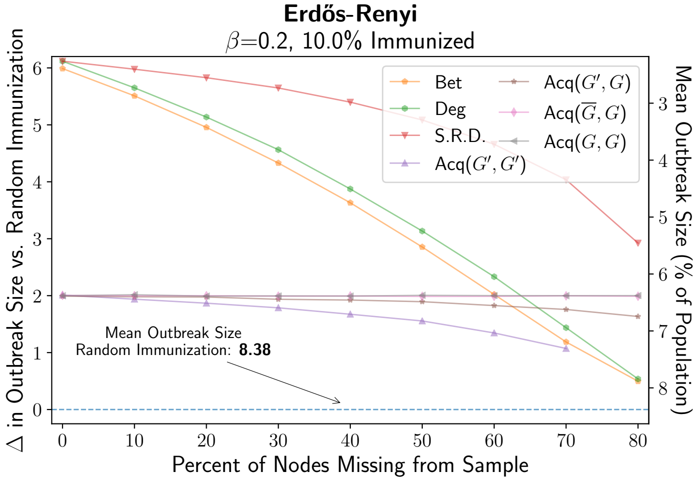

(i)

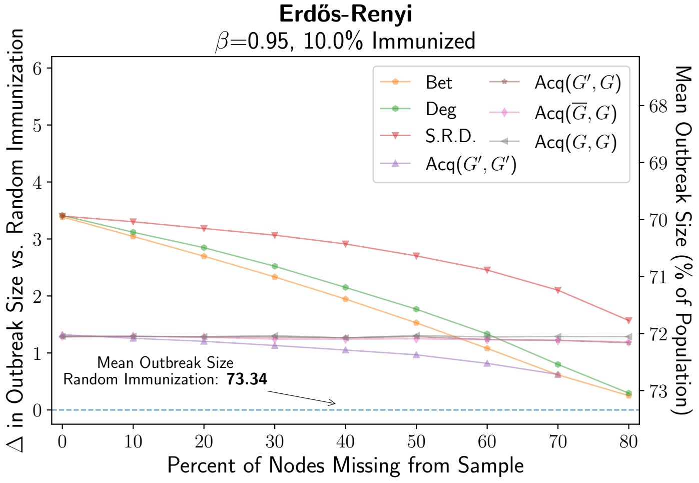

(ii)

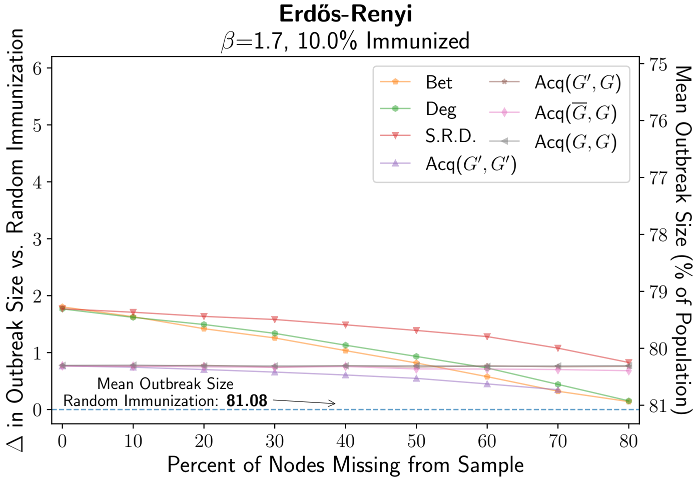

(iii)

Fig A. Relationship between missing data and immunization effectiveness given “true networks” created via an Erdős-Rényi (GNM) [1] process with $N = 1000$ (equal to the Colorado Springs networks in the main text) and $M = 4324$ (equal to the mean number of edges of the Colorado Springs networks in the main text). Implementation and plotting schema identical to Figs 2, 3, and 4 in the main text.

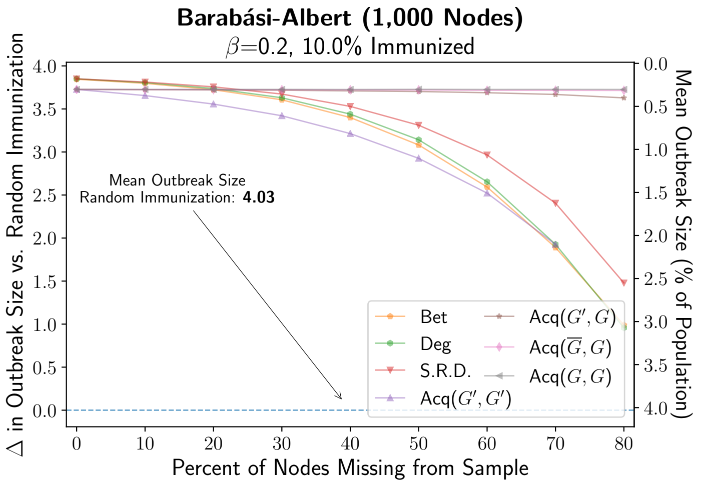

(i)

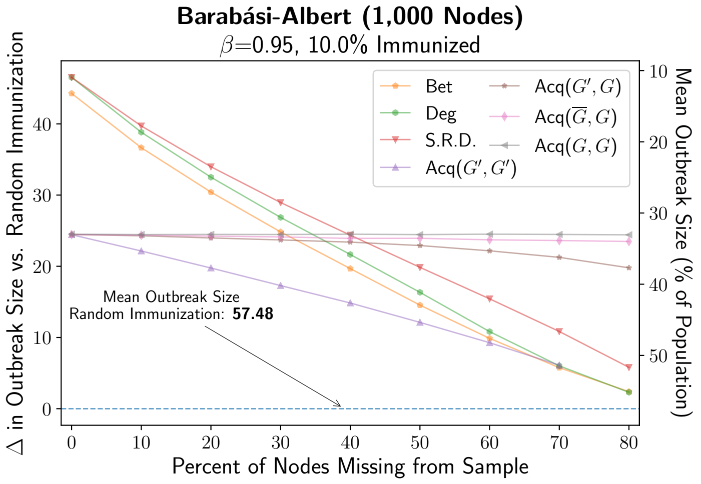

(ii)

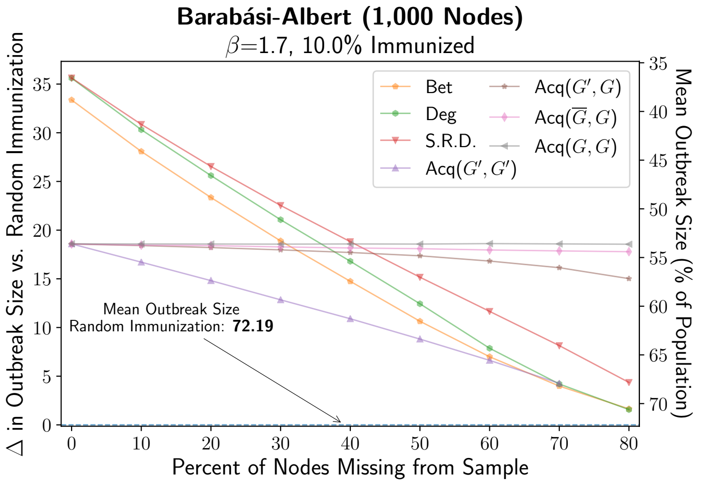

(iii)

Fig B. Relationship between missing data and immunization effectiveness given "true networks" created via a Barabási-Albert [2] process with seed nodes $m_0 = 5$, and $m = 3$ edges added to each additional node (process identical to those tested in [3] but with 1000 nodes for comparability to networks in the main text). Implementation and plotting schema identical to Figs 2, 3, and 4 in main text. Subfigures show the effects of varying the infectiousness parameter β. Note that the y-scale is different between subfigures, as (i) would be unreadable at the appropriate scale for (ii) and (iii)

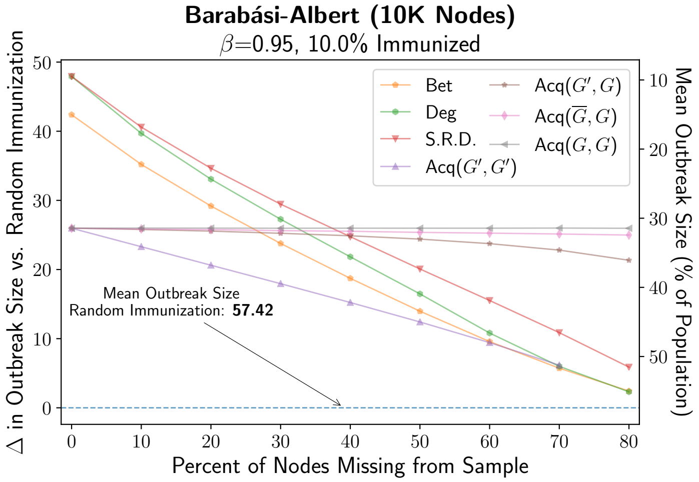

(i)

Fig C. Relationship between missing data and immunization effectiveness given "true networks" created via a Barabási-Albert [2] process identical to that in [3]. Starting with seed nodes $m_0 = 5$, and adding $m = 3$ edges with each additional node until there were 10,000 total nodes. Implementation and plotting schema identical to Figs 2, 3, and 4 in the main text.

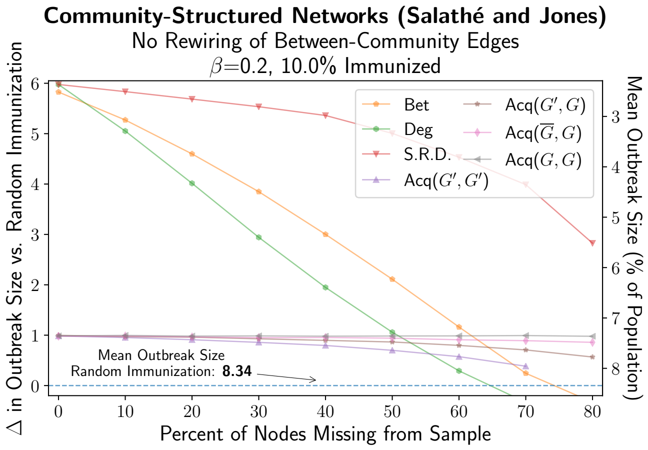

(i)

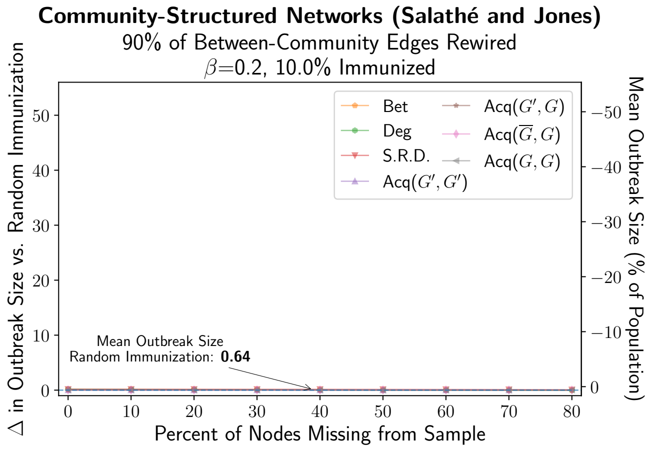

(ii)

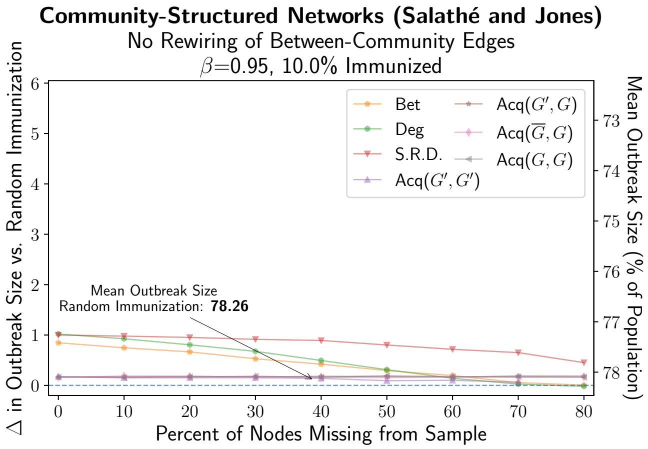

(iii)

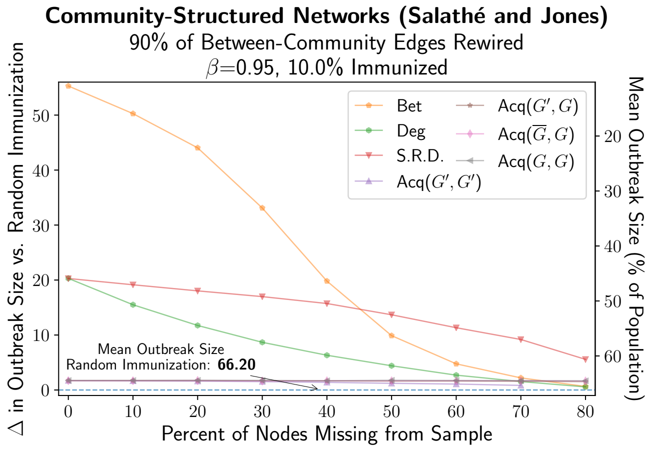

(iv)

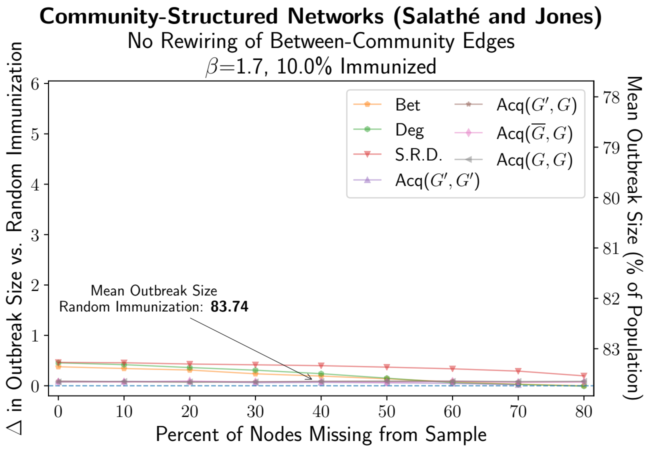

(v)

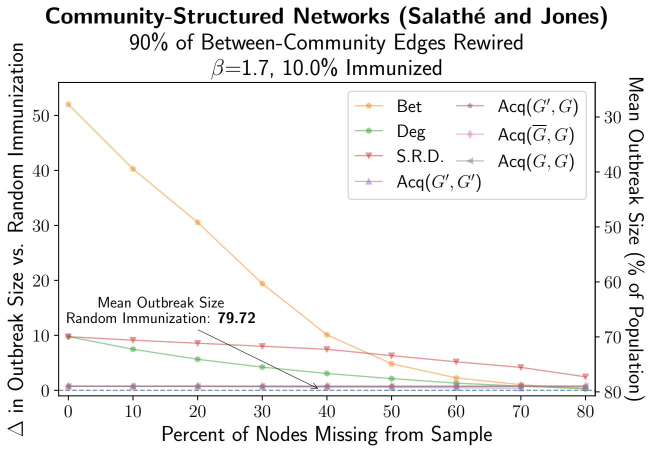

(vi)

Fig D. Relationship between missing data and immunization effectiveness given “true networks” created via the process outlined by Salathé and Jones in Ref. [4]. Implementation and plotting schema identical to Figs 2, 3 and 4 in the main text. Immunization coverage set to 10%. In panels (i),(iii), and (v), networks were created by adding 2000 between-community edges to connect 50 Watts-Strogatz “small-world” networks [5] of size 40 with initial degree 8 and within-community rewiring probability 0.1. This process produces networks with modularity approximately 0.76 (according to the spinglass method, [6]) which, by the same measurement, approximately matches the modularity of the networks with no between-community rewiring used in the paper by Salathé and Jones) [4]. In panel (ii), (iv), and (vi), 90% of these between community edges were re-wired into within-community edges to create networks with modularity approximately equal to 0.93, a value close to the high end of those tested by Salathé and Jones.

2 Supplementary Tests

2.1 Significance Tests for Results of Figs 2-4 in the Main Text

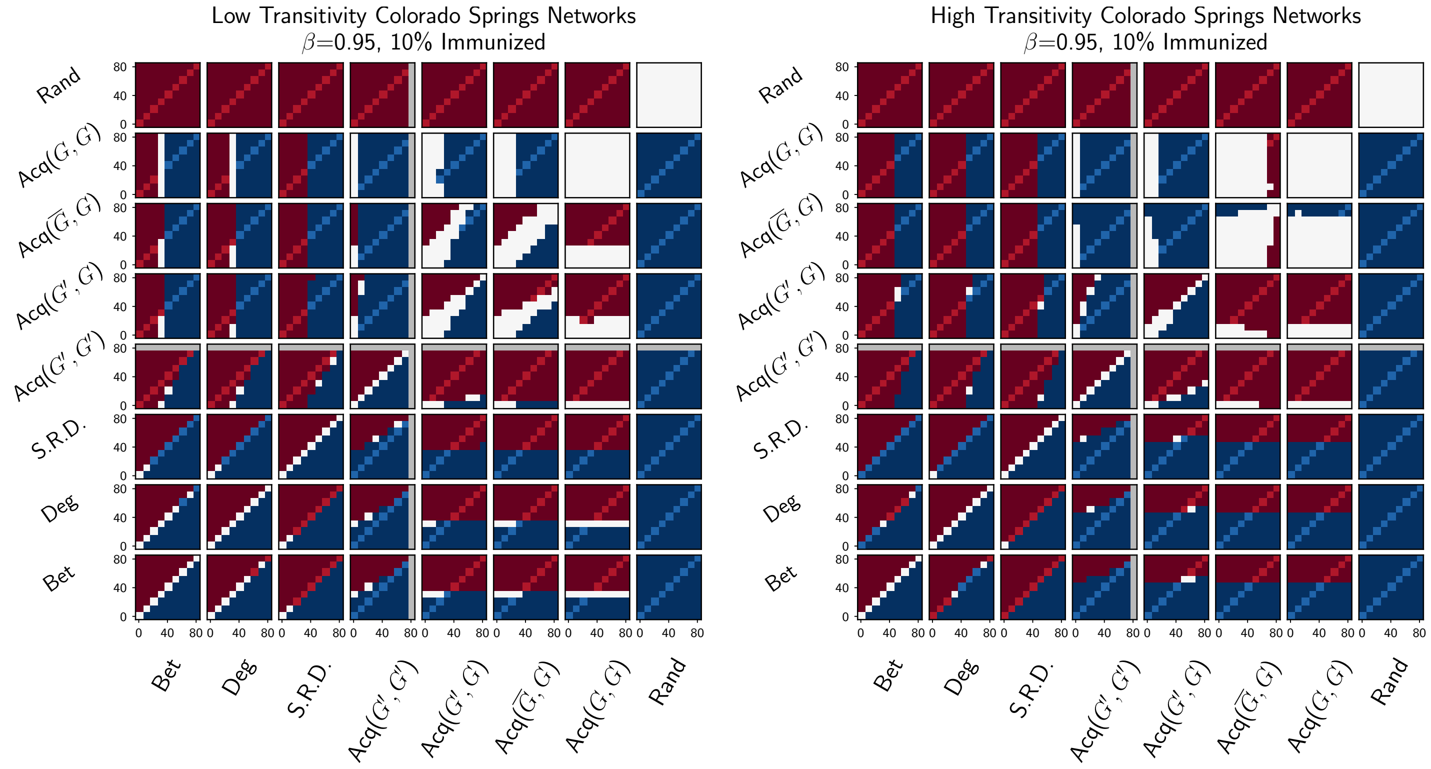

(i) **(ii)**

Fig E. Significance of Comparisons Between Strategies on High and Low Transitivity Networks. We here test the significance of results from Fig 3 of the main text using Bonferroni-corrected t-tests to compare the distributions of mean outbreak sizes. For the subplot at row A, column B, the color at position (i, j) indicates the directionality and significance of the difference in mean outbreak size between strategies A and B, where strategy A is implemented with $i\%$ missing nodes and strategy B is implemented with $j\%$ missing nodes. Blue indicates that strategy A had a lower mean outbreak size (performed better) than strategy B for that parameter set, and that the difference was significant at the $p < \frac{0.001}{M}$ Bonferroni-corrected level, whereas red would indicate the opposite, and white would indicate that their distributions were not significantly different. For these analyses, the number of tests, M in the denominator for the Bonferroni correction is $\frac{(8*9)^2-(8*9)}{2}$, which is equal to the number of comparisons between any two choices of immunization strategy (out of 8) and any two choices for level of missing data (out of 9), where order does not matter, and we do not compare a distribution to itself. The diagonals at positions $i = j$ (equal level of missing data) have been highlighted for increased readability. Positions which are grey were those which involved trials where the strategy did not converge.

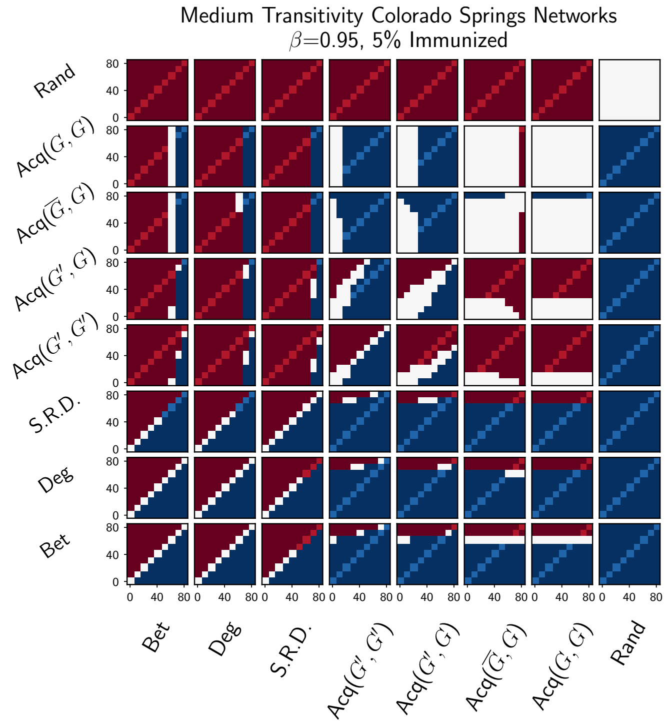

(i)

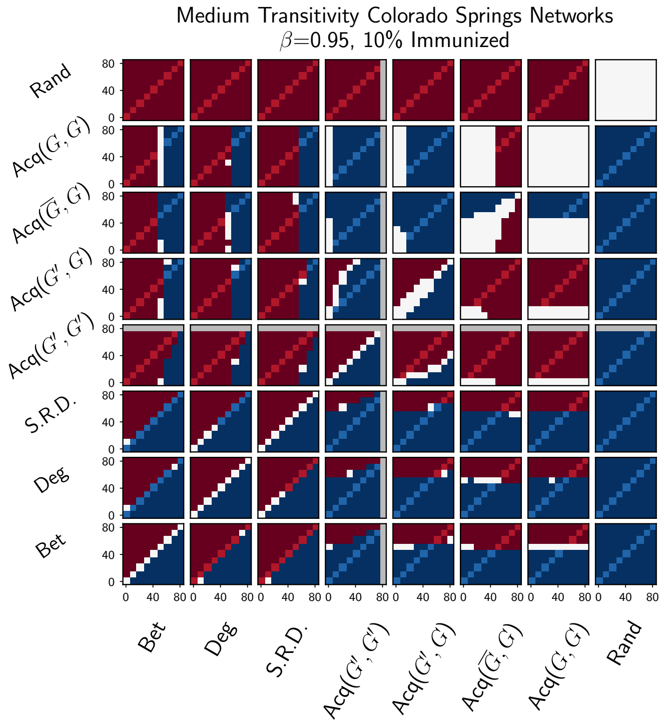

(ii)

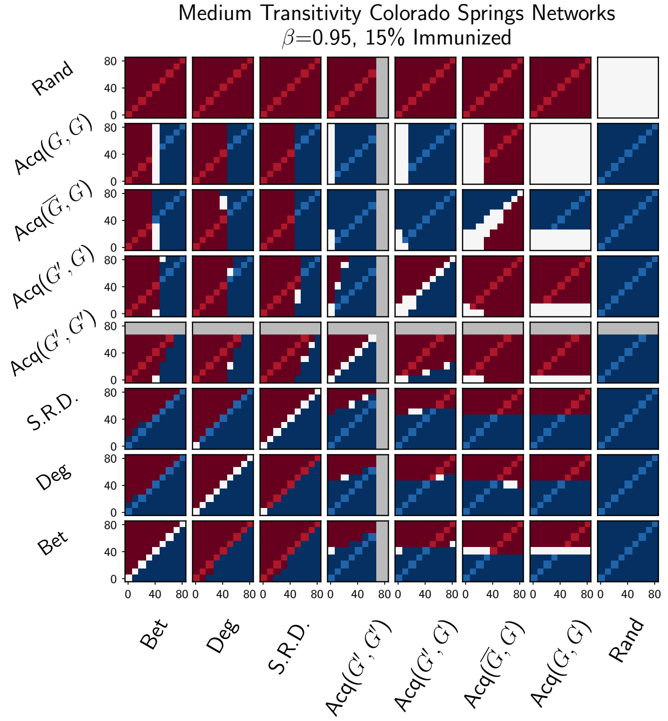

(iii)

Fig F. Significance of Comparisons Between Strategies on Medium Transitivity Networks. These figures correspond directly to the data presented in Fig 2 of the main text. Plotting schema identical to Fig E.

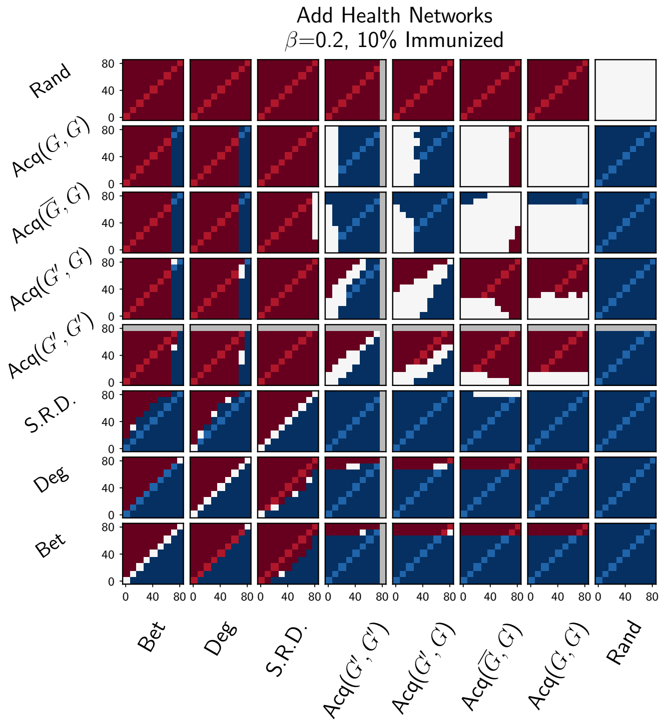

(i)

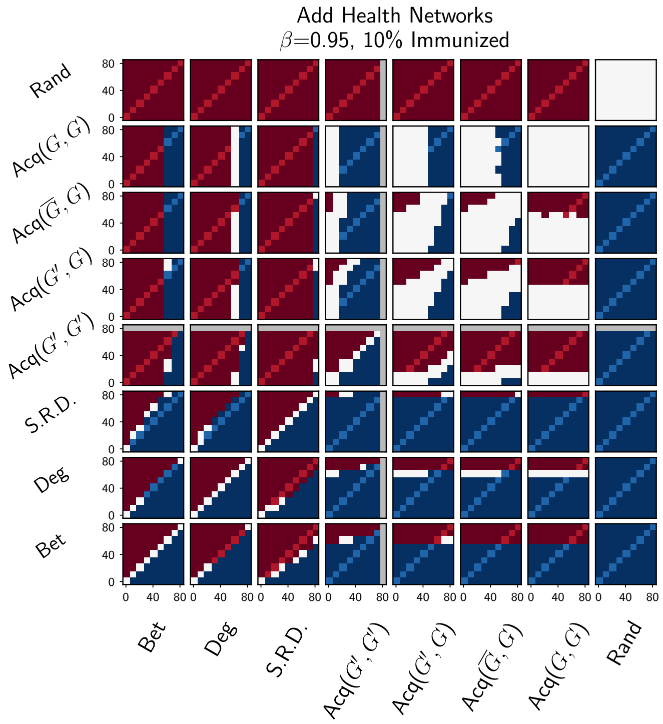

(ii)

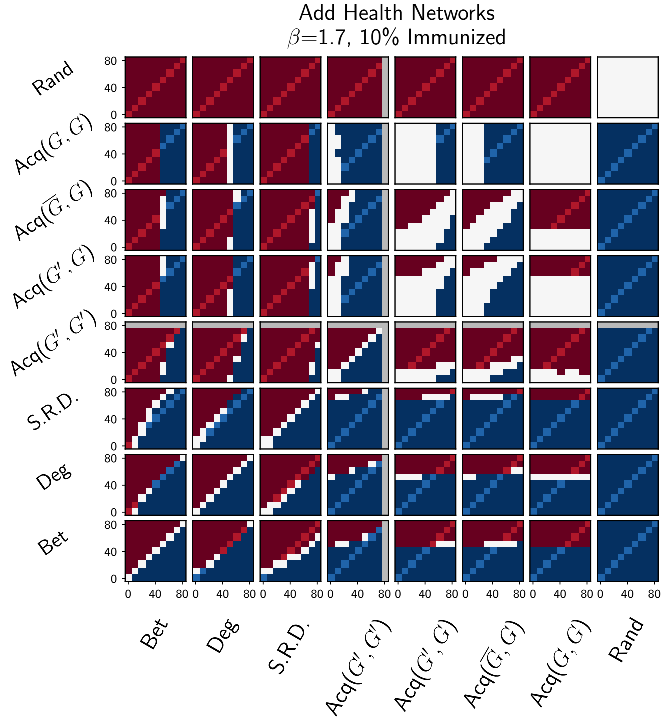

(iii)

Fig G. Significance of Comparisons Between Strategies when varying β on Add Health Networks. These figures correspond directly to the data presented in Fig 4 of the main text. Plotting schema identical to Fig E.

2.2 Analysis of variability of outbreak sizes

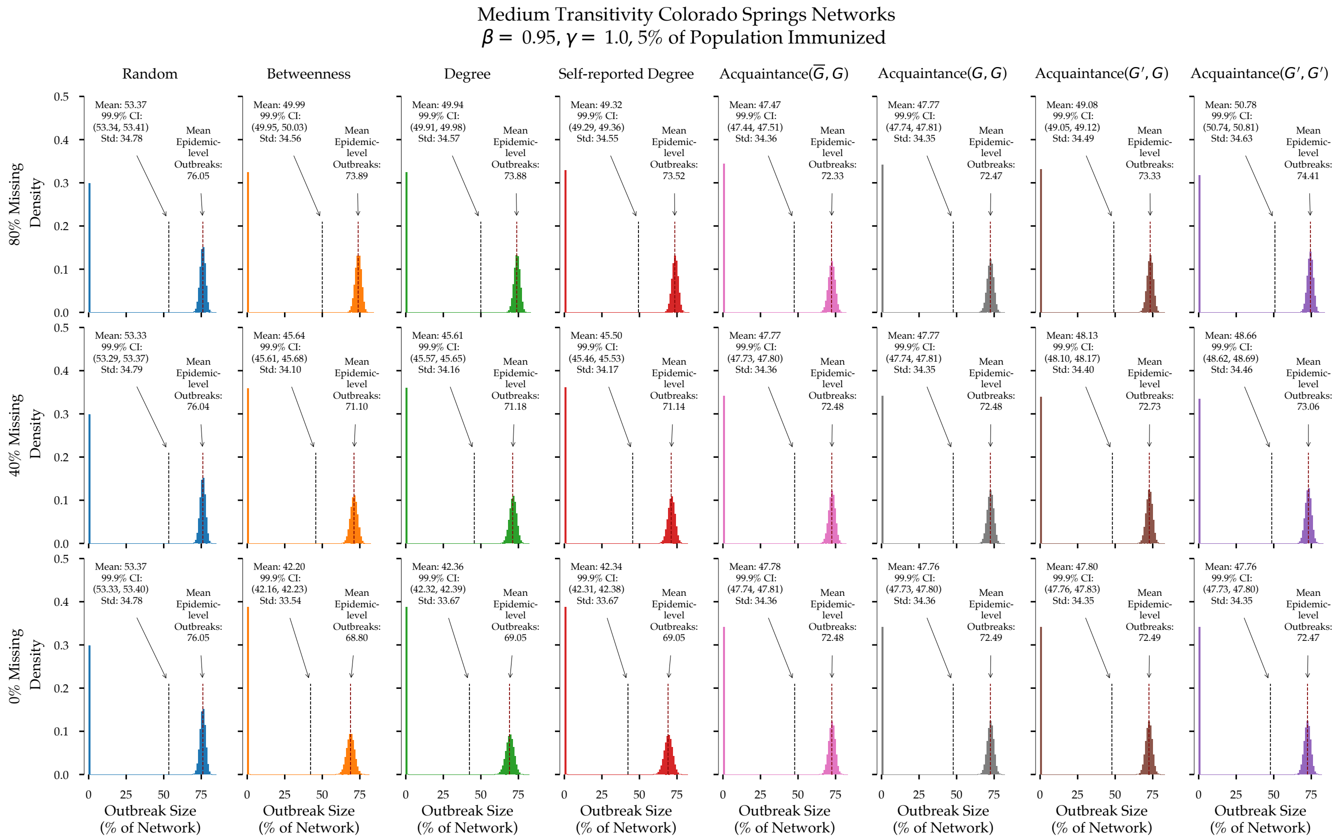

Fig H. Distributions of Individual Simulations for Fig 2A. The distributions shown here represent the outbreak sizes of all 10 million individual simulations for a given parameter set, spread out over 5000 networks generated from the same mechanism with similar characteristics. The data underlying this figure corresponds to Fig 2A in the main text (however, in the main text the distribution used is the distribution of the mean outbreak sizes for each network, whereas the distributions shown here are the pooled distribution of all individual outbreak sizes). The grey dotted line in the center of each distribution is the mean, which is annotated with its exact value, as well as a 99.9% confidence interval, and the standard deviation. Note that despite the extreme variability (shown by the standard deviation), the standard errors, and consequently our confidence bounds, are low because of the high number of simulations. Additionally, for comparability with other targeted immunization literature which report only outbreak sizes which exceed some "epidemic-level" threshold [4, 7], we also report the mean of the subset of outbreaks above 1% of the network size in each subplot as the annotated maroon line. The distributions are represented by histograms with frequency density on the y-axis and bin width of 1% (of network size), so that the height of the first bin is equal to the fraction of simulations where the contagion died out without infecting at least 1% of the network. It is worth noting that for many set of parameters, the mean value of an outbreak size distribution is approximately equal to: The probability that a given outbreak is above the epidemic threshold, multiplied by the mean of the second mode; and to the squared value of the relative outbreak size [8].

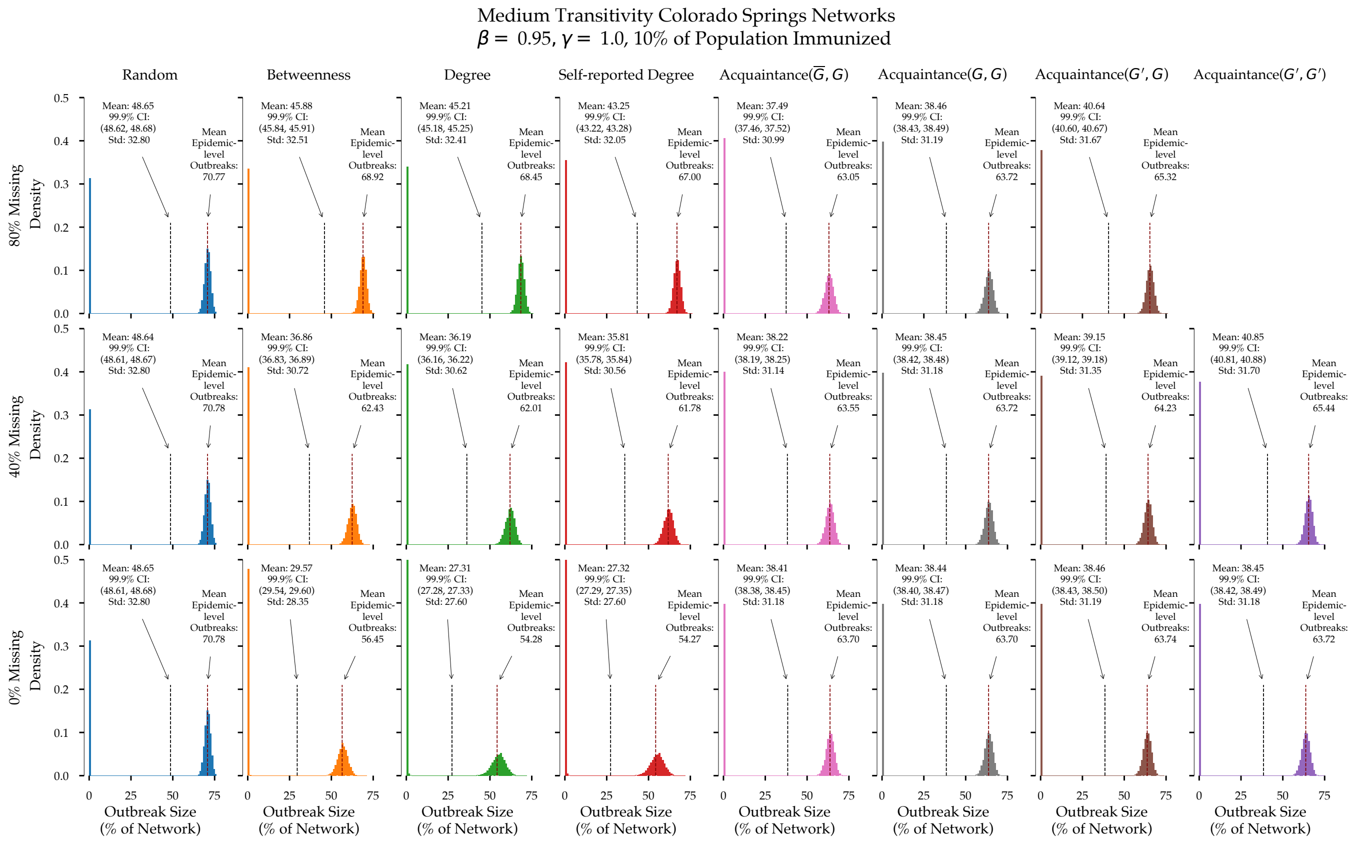

Fig I. Distributions of Individual Simulations for Fig 2B.

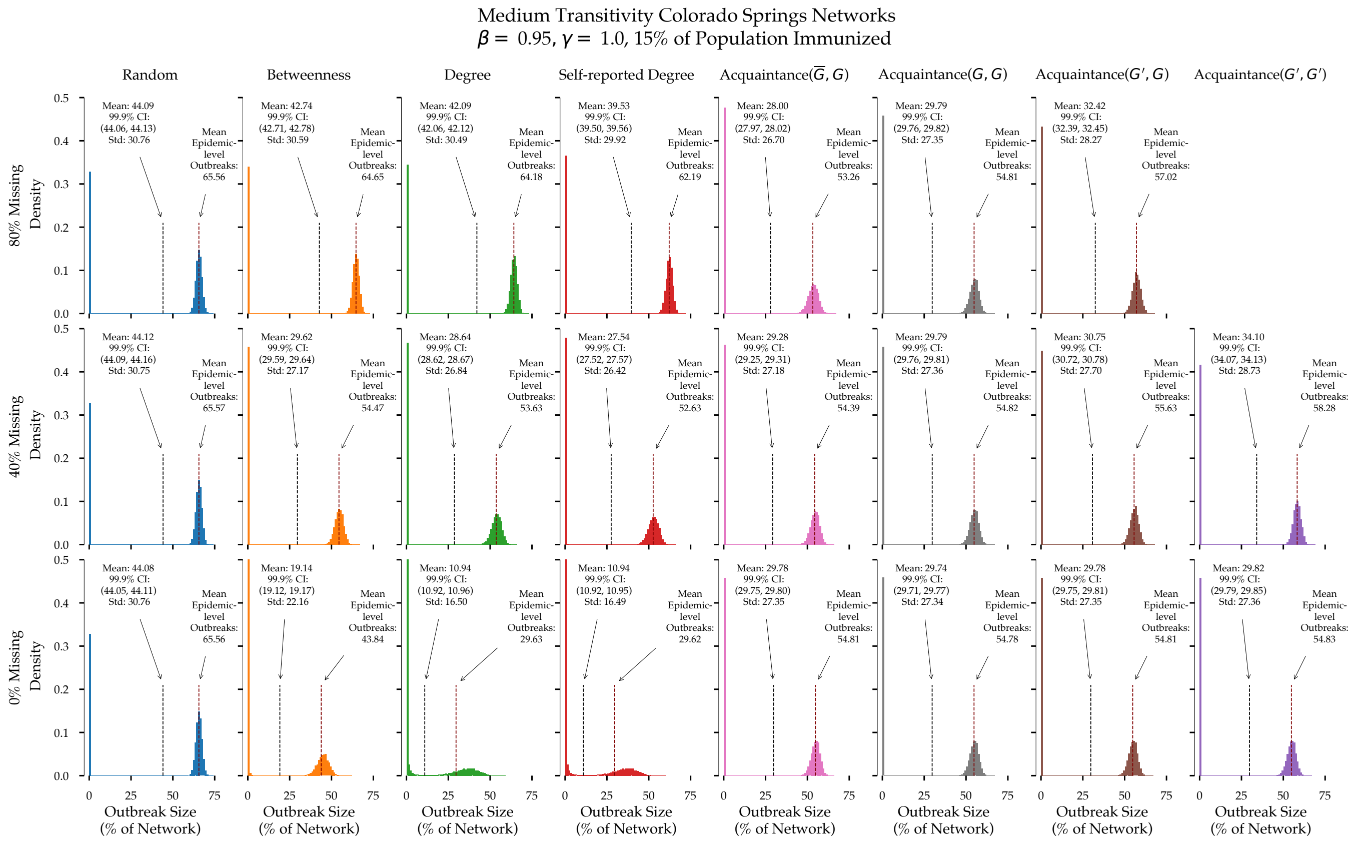

Fig J. Distributions of Individual Simulations for Fig 2C.

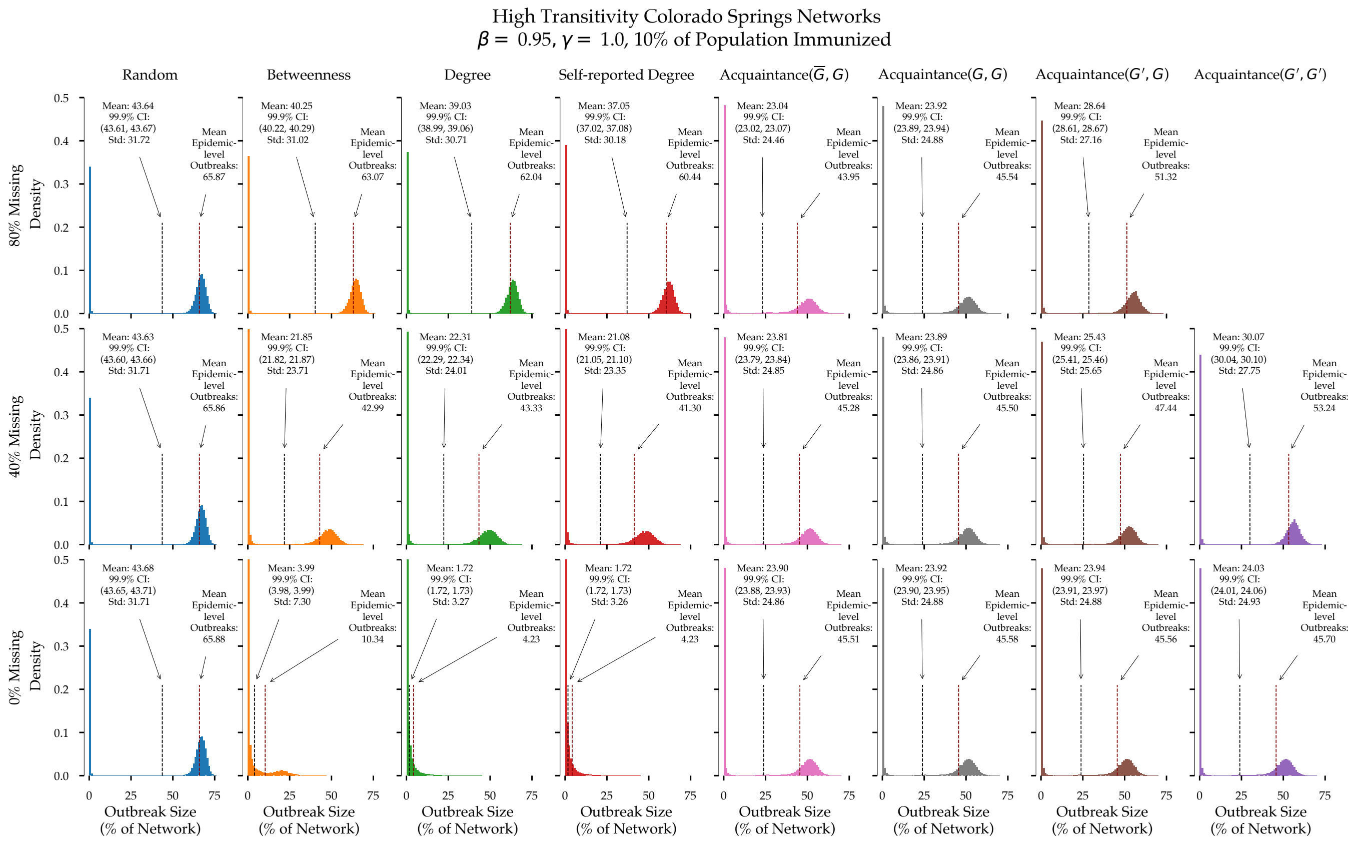

Fig K. Distributions of Individual Simulations for Fig 3A.

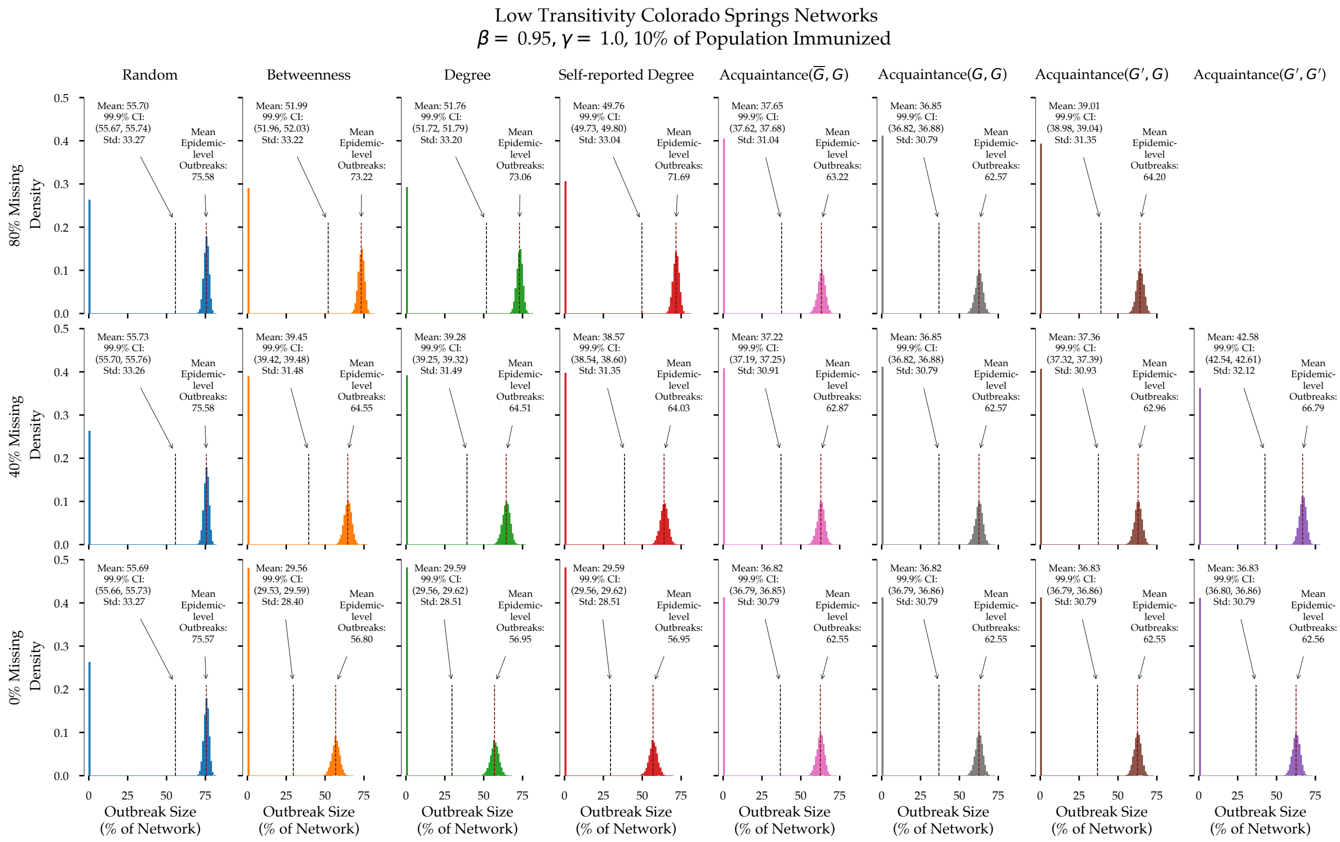

Fig L. Distributions of Individual Simulations for Fig 3B.

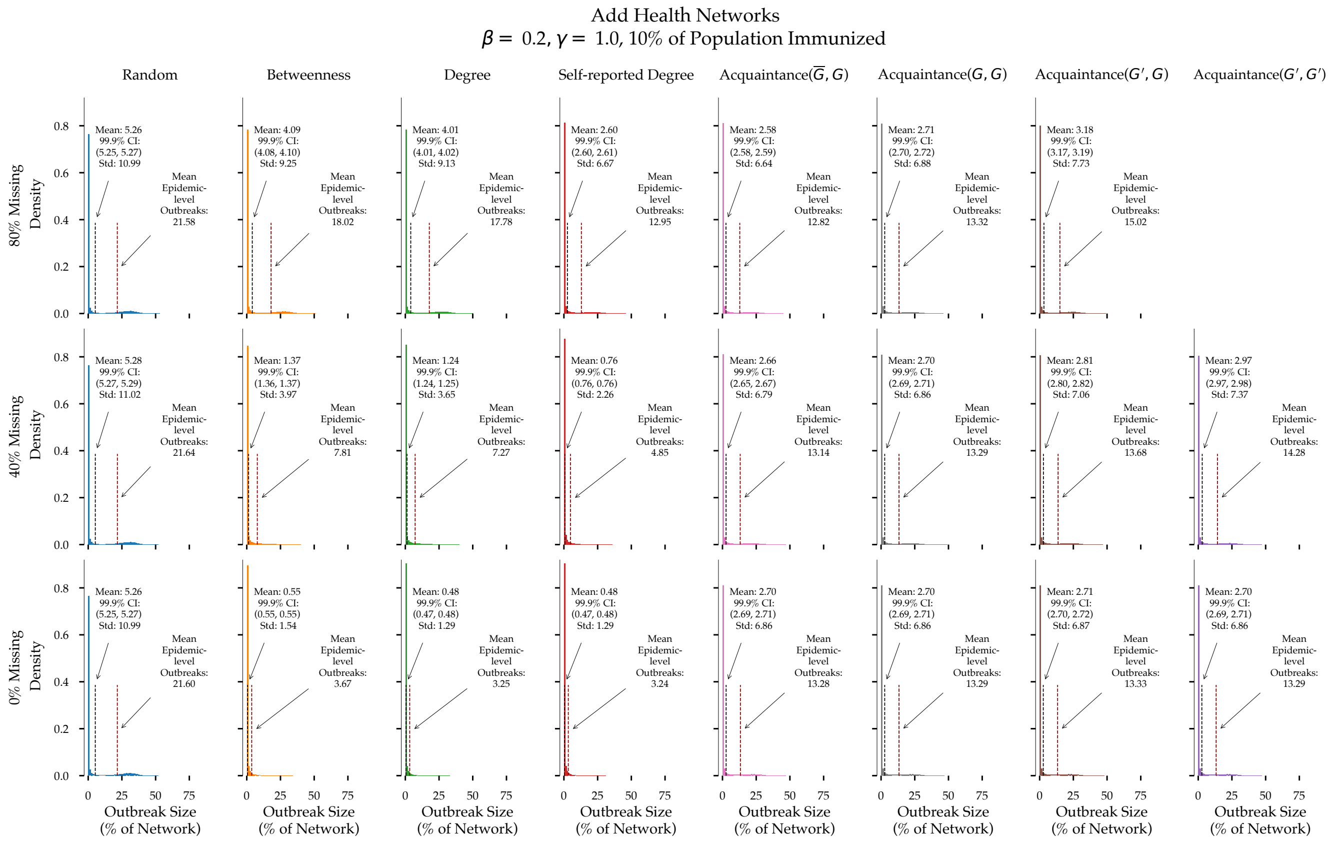

Fig M. Distributions of Individual Simulations for Fig 4A.

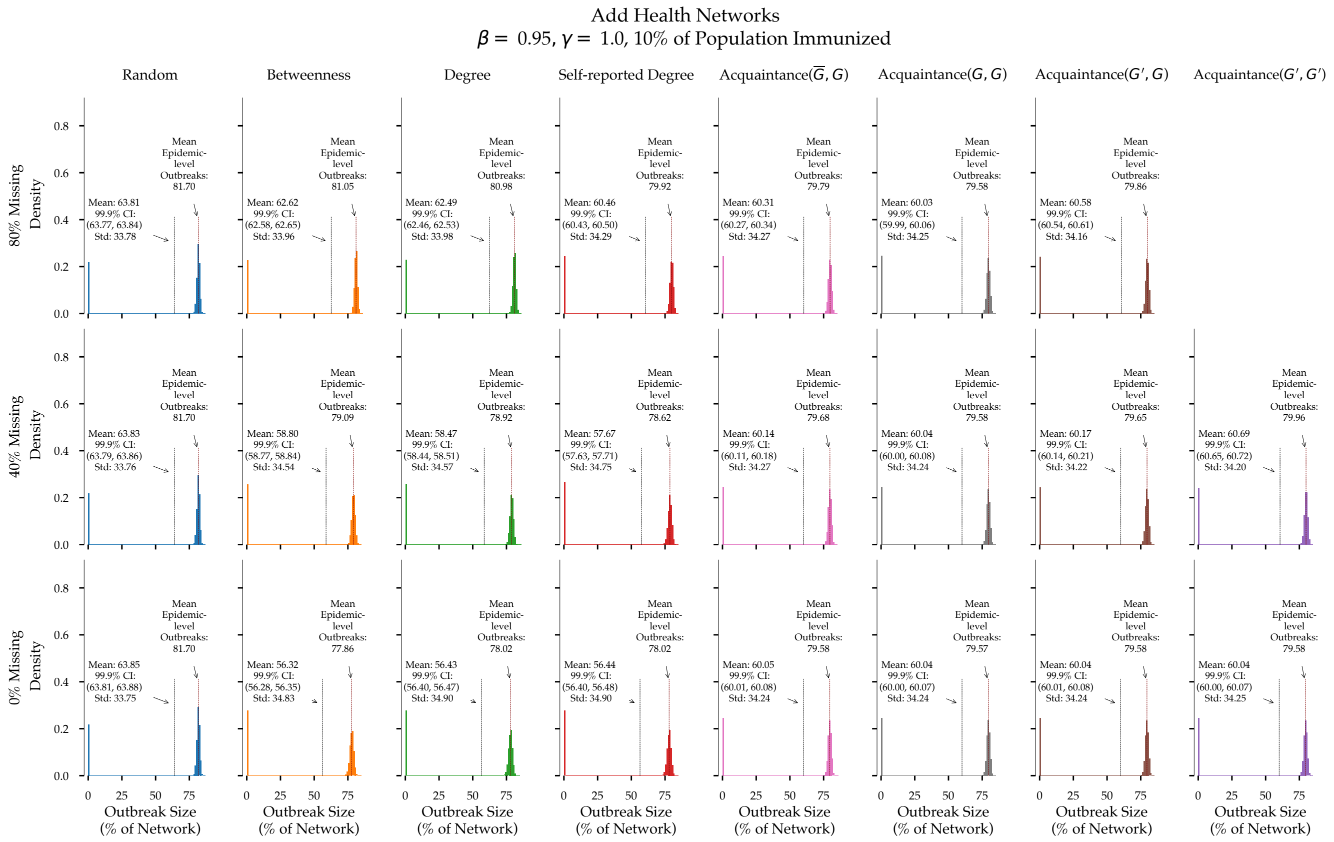

Fig N. Distributions of Individual Simulations for Fig 4B.

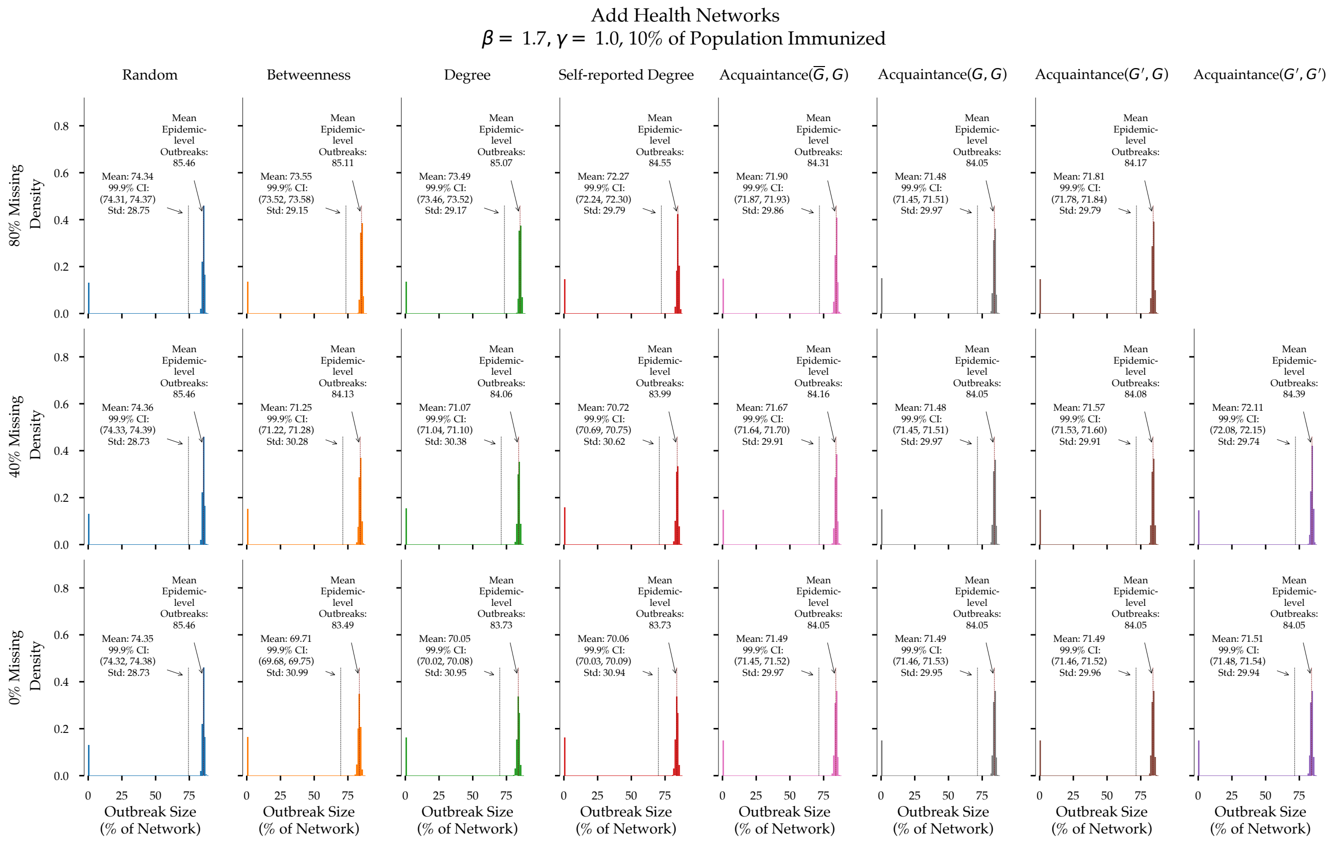

Fig O. Distributions of Individual Simulations for Fig 4C.

2.3 Full Parameter Space for Networks in the Main Text

2.3.1 Low Transitivity Colorado Springs Networks

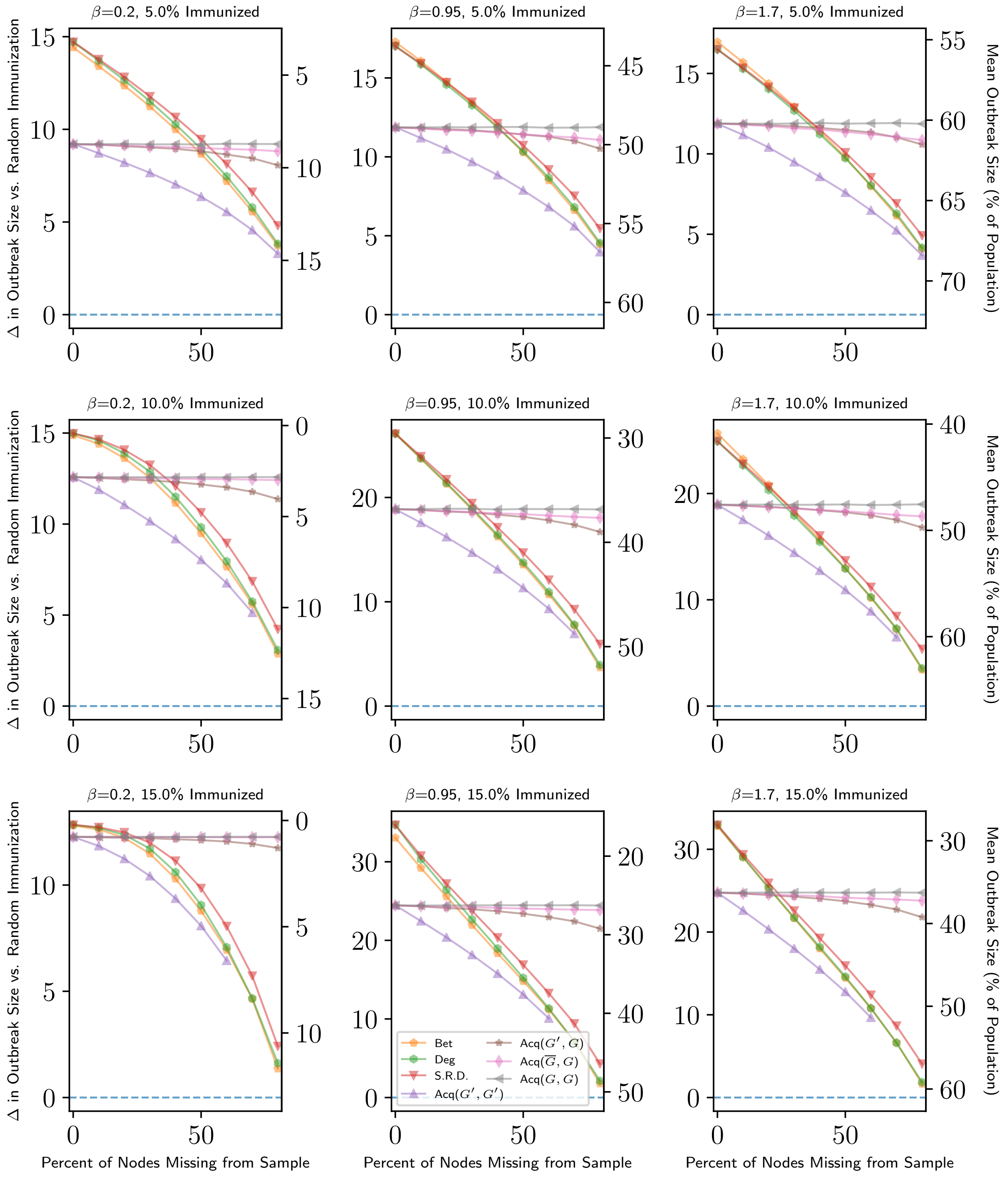

Fig P. Parameter Sweep on Low Transitivity Colorado Springs Networks.

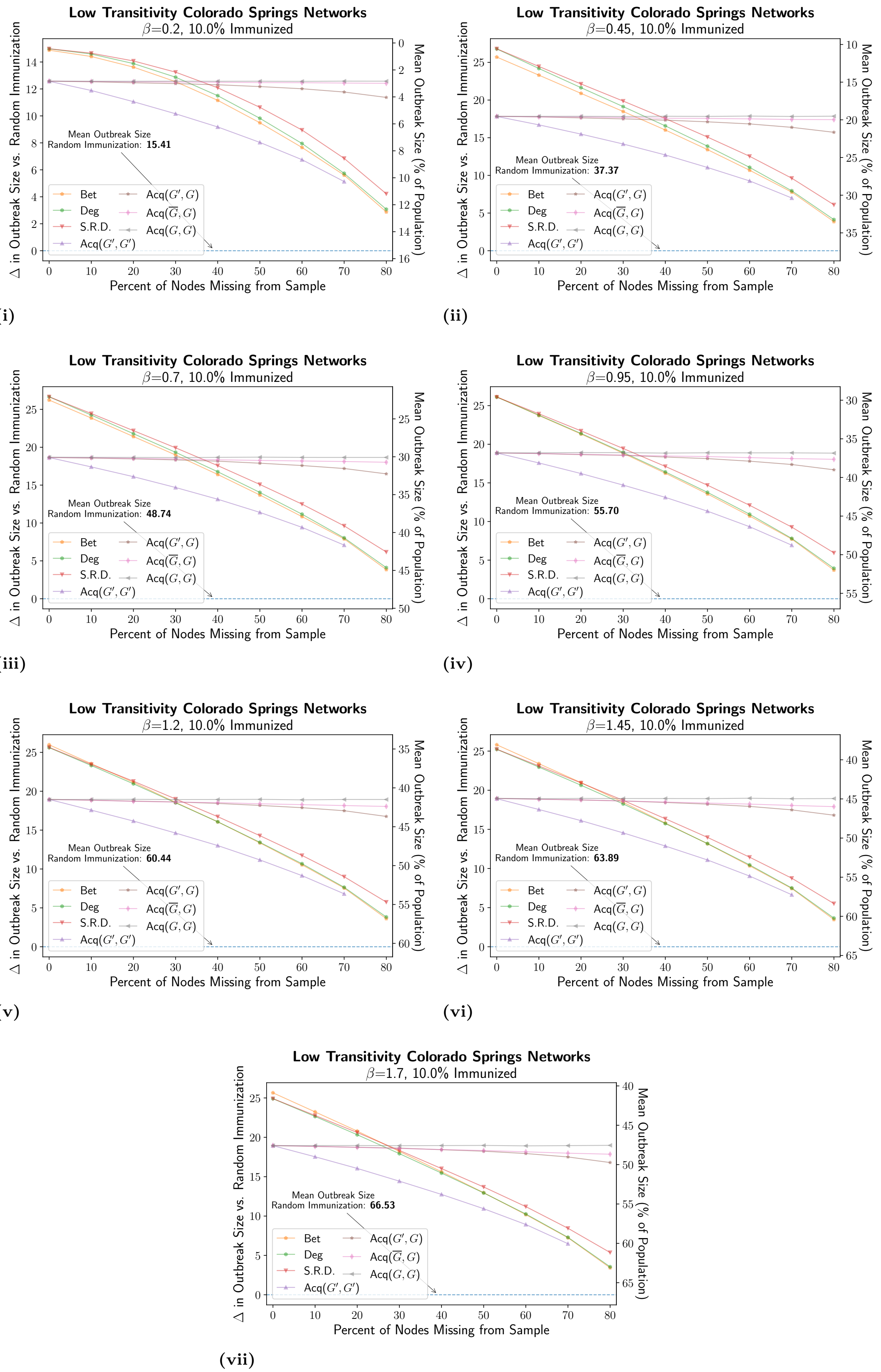

Fig Q. Fine-Grained Sweep of β for Low Transitivity Colorado Springs Networks at 10% Immunization.

2.3.2 Medium Transitivity Colorado Springs Networks

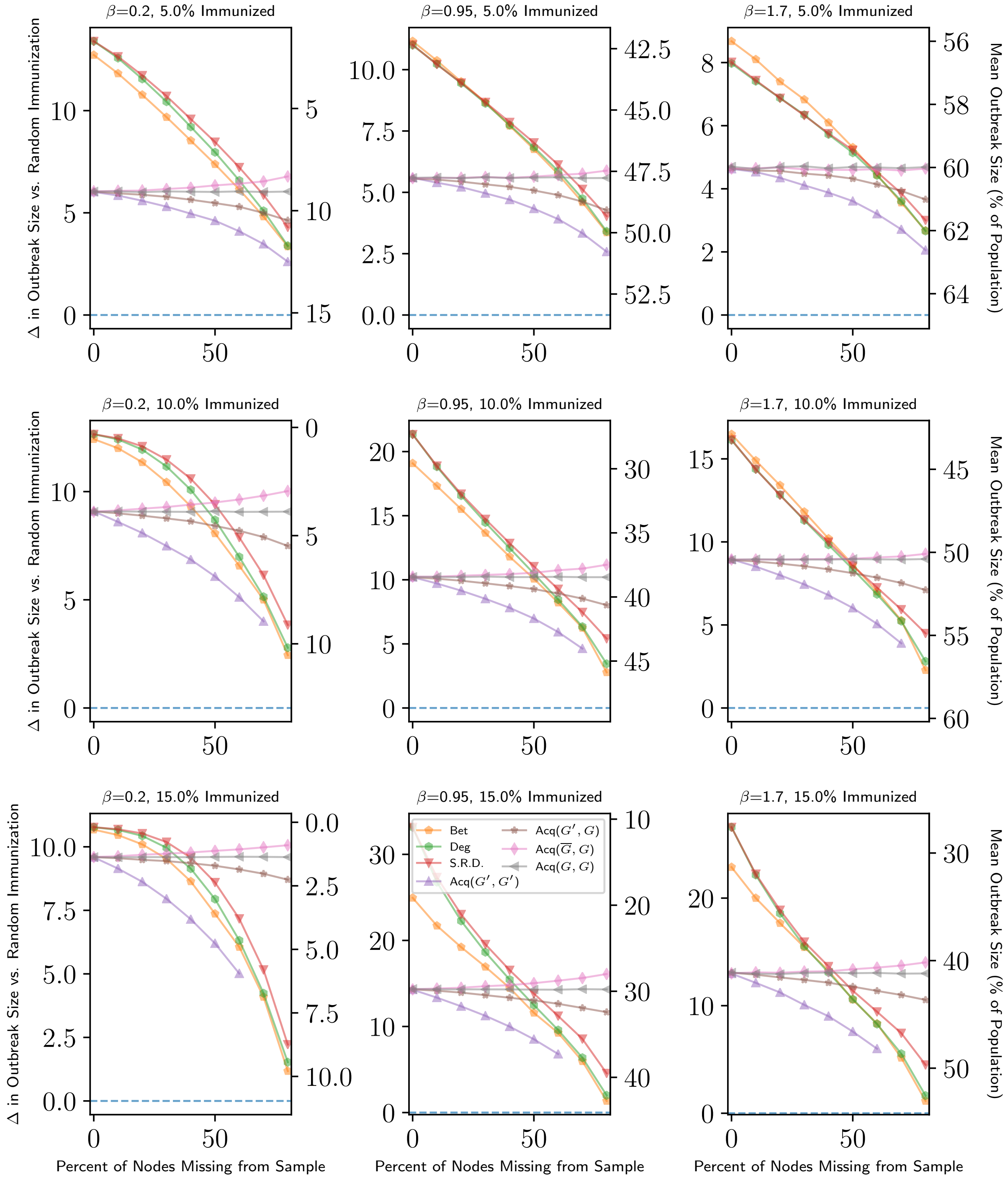

Fig R. Parameter Sweep on Medium Transitivity Colorado Springs Networks.

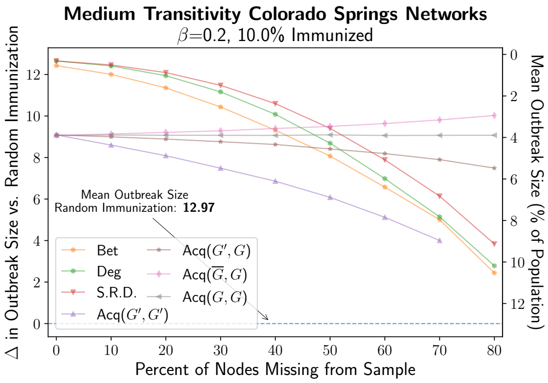

(i)

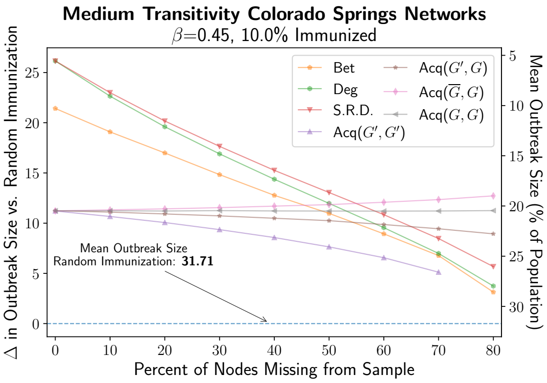

(ii)

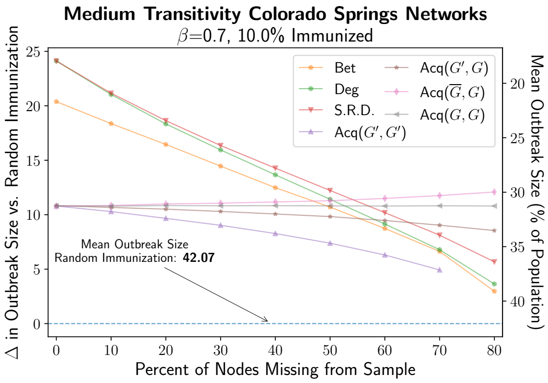

(iii)

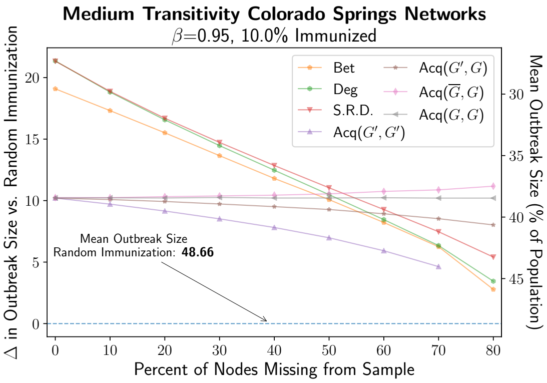

(iv)

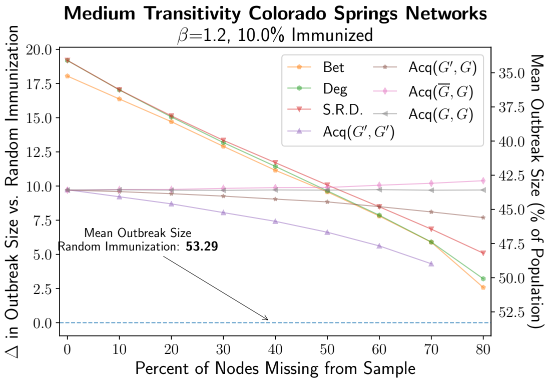

(v)

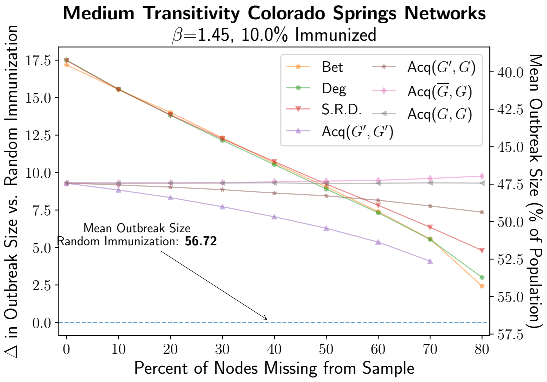

(vi)

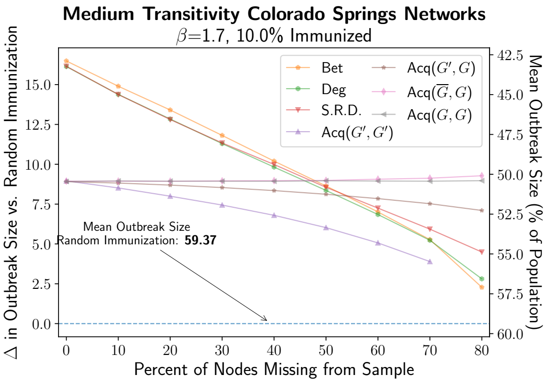

(vii)

Fig S. Fine-Grained Sweep of β for Medium Transitivity Colorado Springs Networks at 10% Immunization.

2.3.3 High Transitivity Colorado Springs Networks

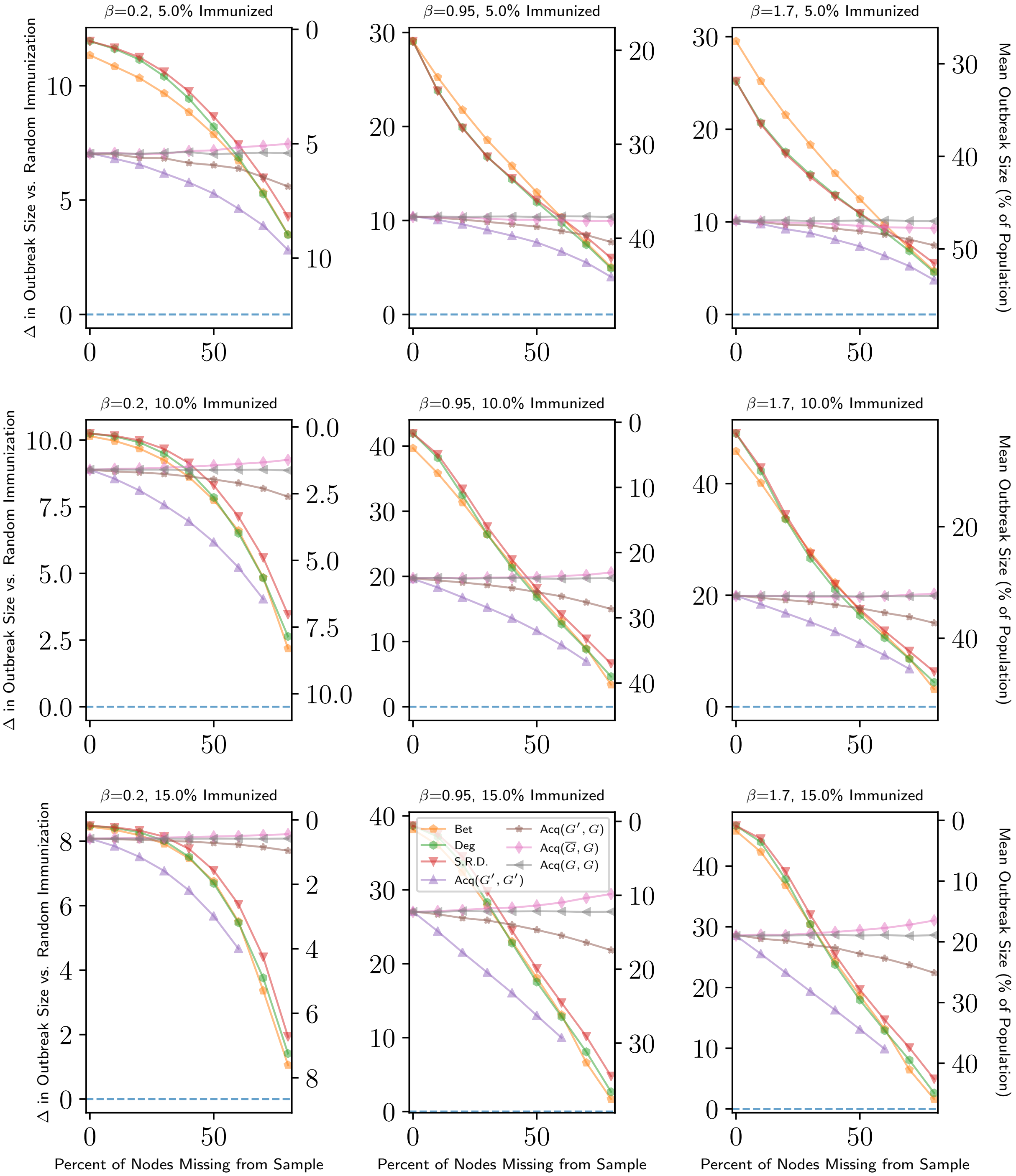

Fig T. Parameter Sweep on High Transitivity Colorado Springs Networks.

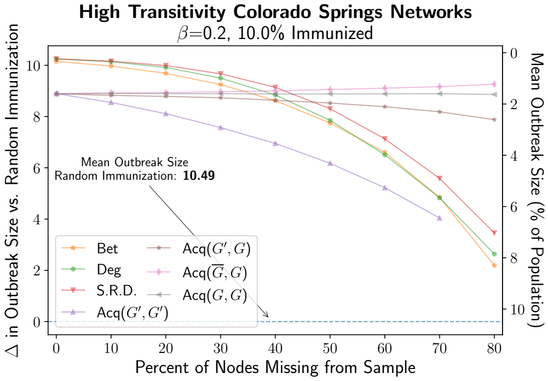

(i)

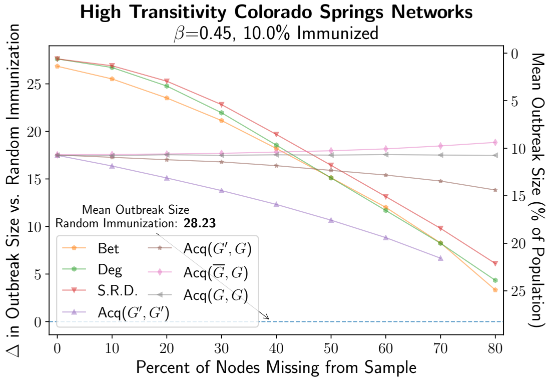

(ii)

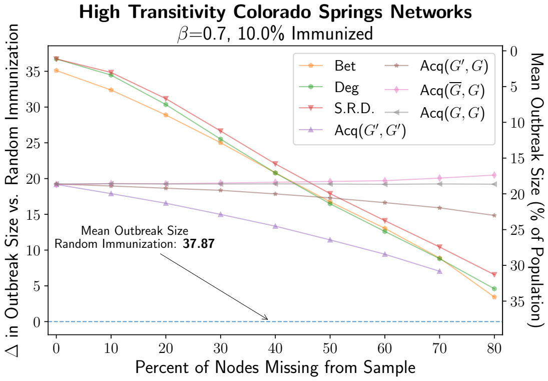

(iii)

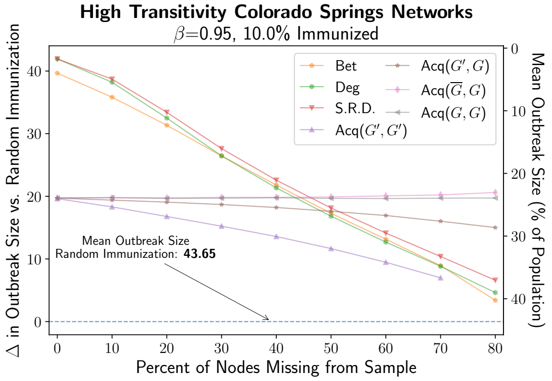

(iv)

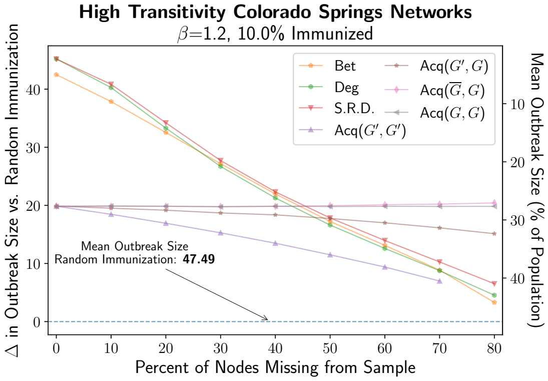

(v)

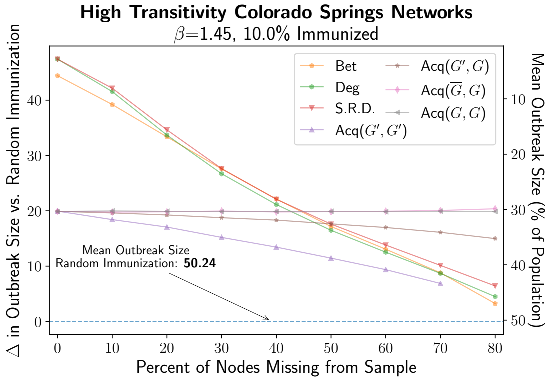

(vi)

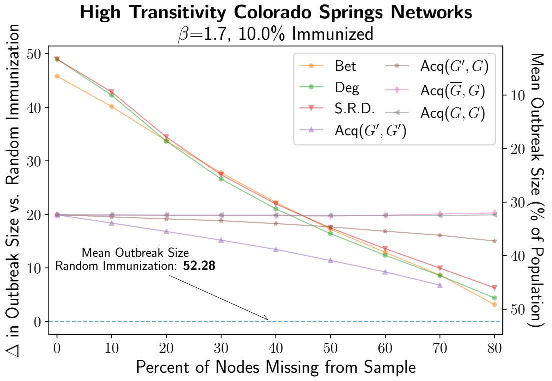

(vii)

Fig U. Fine-Grained Sweep of β for High Transitivity Colorado Springs Networks at 10% Immunization.

2.3.4 Add Health Networks

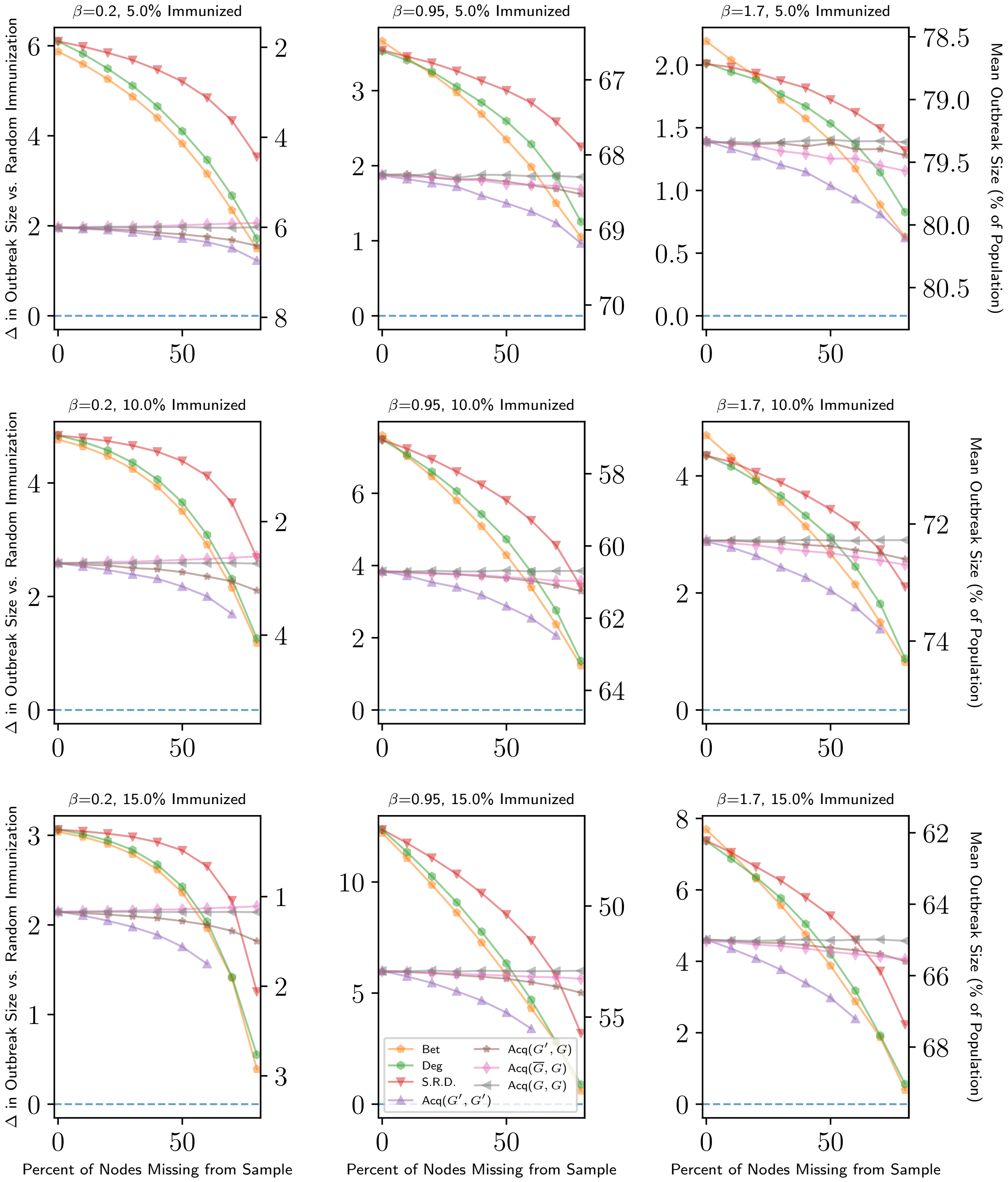

Fig V. Parameter Sweep on Add Health Networks.

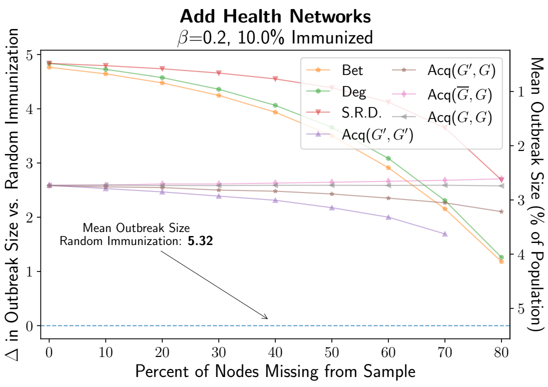

(i)

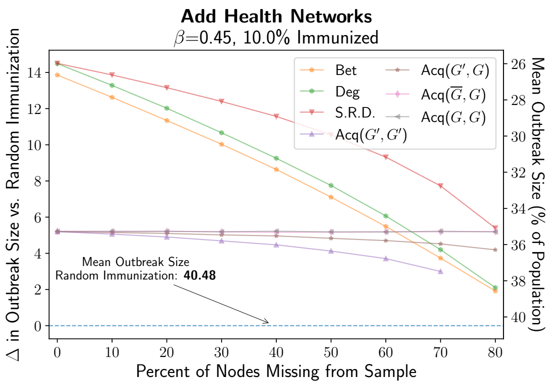

(ii)

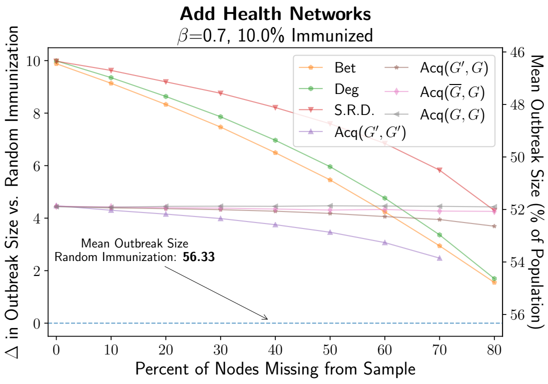

(iii)

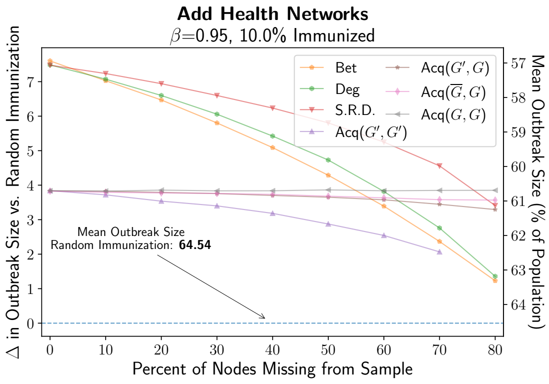

(iv)

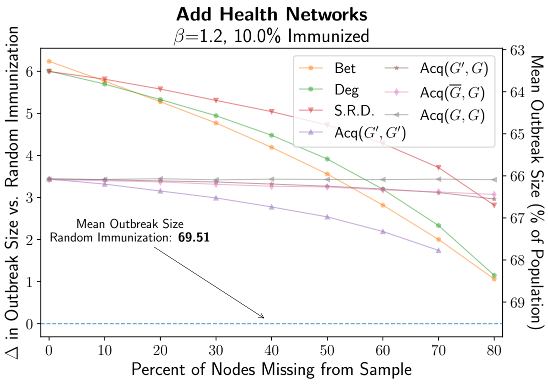

(v)

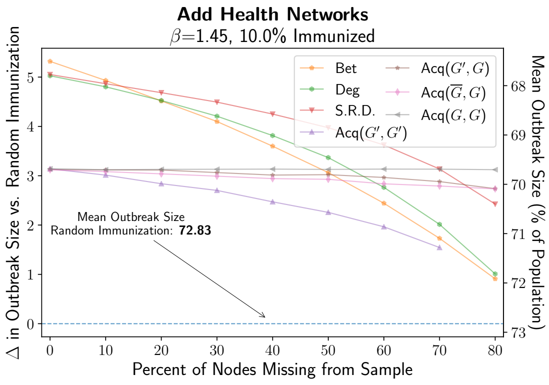

(vi)

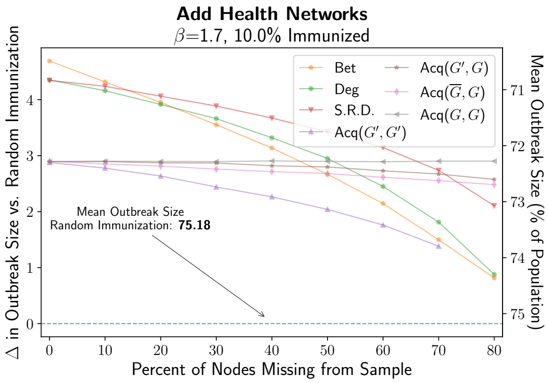

(vii)

Fig W. Fine-Grained Sweep of β for Add Health Networks at 10% Immunization.

3 Additional Limited Analyses

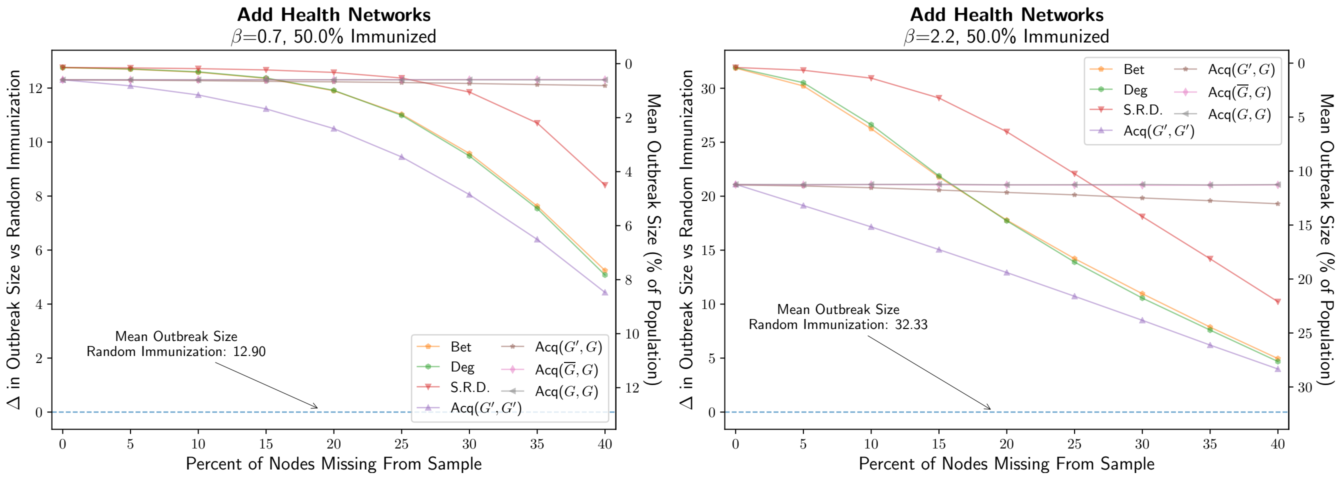

(i) (ii)

Fig X. Results from Add Health Networks at a higher value of β with substantially higher immunization coverage (50%). We offer a limited look at how these strategies might perform if implemented in a scenario like an influenza outbreak at a school, where immunization units are available in much higher abundance. The measured value of R_0 for this network with $\beta = 2.2$ was 4.00, which is more infectious than the typical strain of influenza. The outbreak size with no immunization was 89.31% of the network. Note that since the majority of these strategies may only immunize nodes in their observed network (exceptions include Acq(G, G), Acq$(\overline{G}, G)$ and Acq(G', G)), that they are by definition limited in their immunization coverage by the percent of the network data they have available. At 40% missing data, the observed network that the strategies are using is only 60% of the size of the full network, and thus, immunizing 50% of the network using only this 60% means immunizing 5 out of every 6 nodes available. Choosing which 5 out of every six nodes by degree immunization can still reduce the size of outbreaks, but at that point, for many networks, the “targeted” nodes are not much different than randomly chosen nodes.

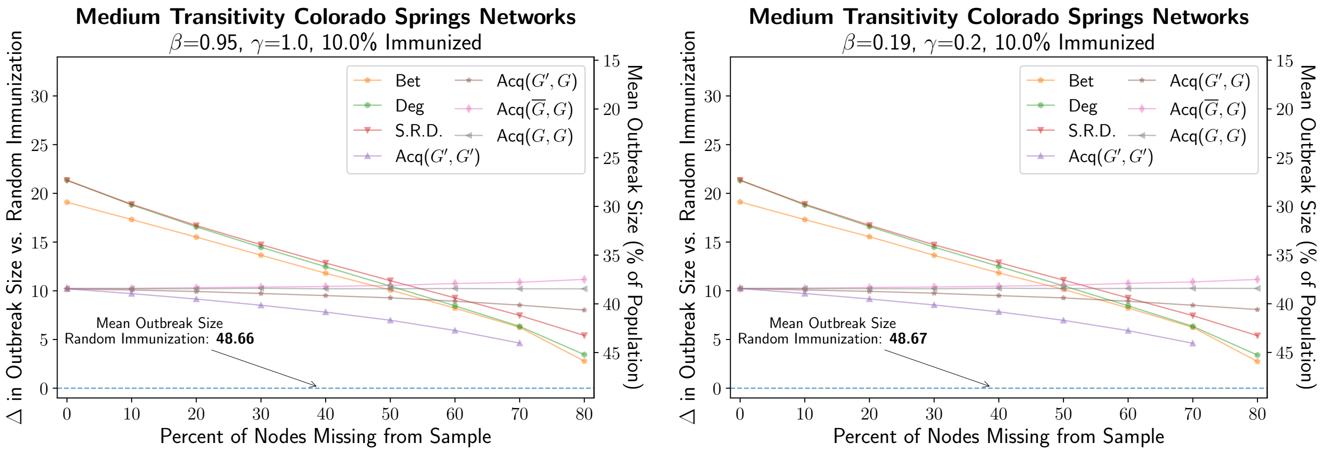

(i) (ii)

Fig Y. Comparison of simulations with equal ratios of SIR parameters β and γ. Note that despite the considerably different scales of the two sets of parameters, the results are indistinguishable from each other. This illustrates that for SIR dynamics on networks, although β and γ are important mechanistically to the simulation model, only the average transmission probability T matters when considering final outbreak size (as opposed to time-related measurements). The value T is at times approximated by the ratio $\frac{\beta}{\gamma}$ [4] but actually given by $\frac{\beta}{\beta+\gamma}$ [8, 9].

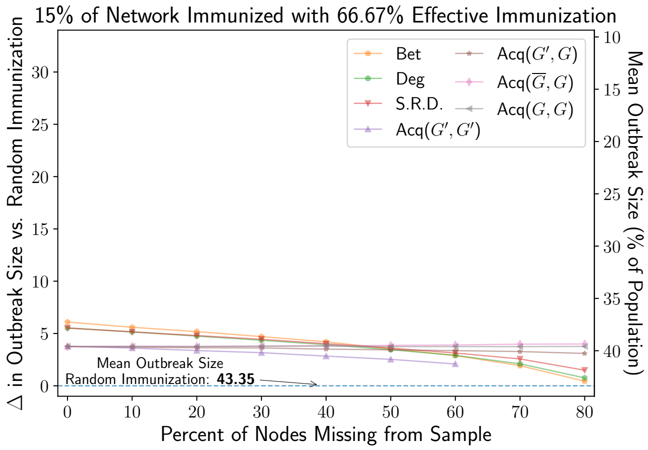

(i)

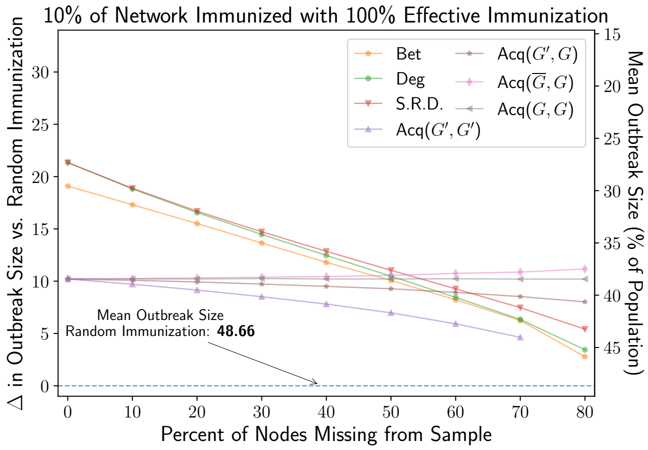

(ii)

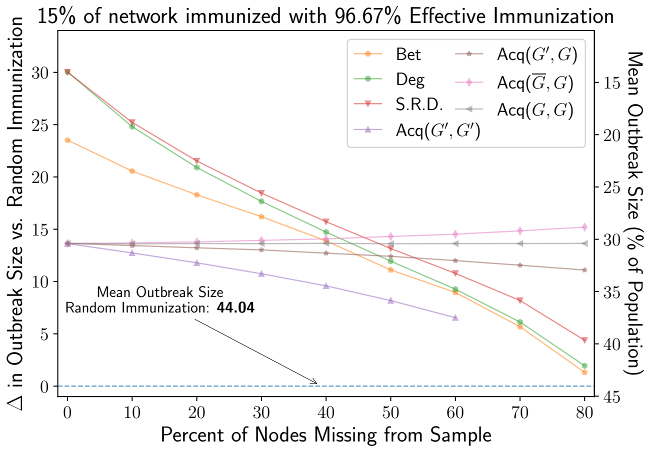

(iii)

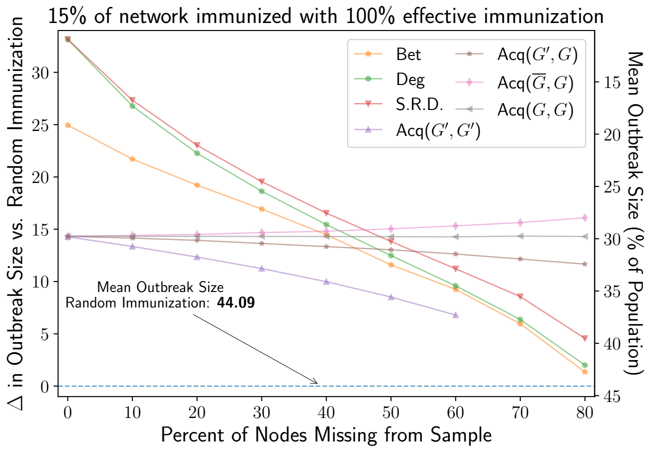

(iv)

Fig Z. Exploration of the effect of imperfect immunization: Medium Transitivity Colorado Springs Networks. Here we offer a limited analysis of the effect of removing an additional assumption from our model: the assumption of 100% effective immunization. In panels (ii) and (iv), as in in all other analyses throughout this paper, 100% nodes targeted by a given strategy were immunized in the simulations for that strategy. In panels (i) and (ii) however, after nodes are targeted by a given strategy for immunization, each targeted node has a given chance, e, that they will not be immunized. In panel (iii), this probability is $\frac{1}{30}$ (i.e. the immunization is 96.67% effective). In panel (i) this probability is $\frac{1}{3}$ (i.e. the immunization is 66.67% effective). Since for panel (i), 15% of the network is targeted, but on average only two-thirds of them will be immunized effectively, on average, the simulations for panels (i) and (ii) will have the same number of nodes immunized. In future work, to model a specific kind of intervention, more complex systems could be used to decide whether an immunization is effective for a particular targeted node.

4 Discussion of Results in the Supporting Information

While the effectiveness of the different immunization strategies certainly differed between the network types and parameter sets, in general, we find that the supplementary tests support the conclusions discussed in the main text. In short, all targeted strategies perform better than random immunization even at high levels of missing data, the global strategies perform better than any variant of acquaintance immunization except at high levels of immunization, and strategies which take into account the fact that they have incomplete data and correct for it (acquaintance immunization and self-reported degree) can maintain their performance even at high levels of missing data.

For every type of network, transitivity level, immunization coverage, infection rate, and recovery rate tested, every single targeted immunization strategy is as good or more effective than random immunization. The one partial exception to this rule is shown in Fig D where degree and betweenness immunization are matched by random immunization at the highest level of missing data for some input parameters. However, this is likely an artifact of the mechanistic construction process of the Salathé and Jones networks, where prior to the random edges added between communities, all nodes had a fixed degree of 8 and were within one or two steps of all other nodes in their (small-world) community. The random edges added between communities create some nodes central to the network by increasing the degree and betweenness of the nodes they are added to, however, the majority of nodes remain mostly homogeneous in those measures.

An interesting finding of the tests involving other network types is that the positive effect that missing data has on the $(\overline{G}, G)$ variant of acquaintance immunization is not present. For the Erdős-Renyi, Barábasi-Albert, and Salathé-Jones networks, the $(\overline{G}, G)$ variant does maintain its efficacy even at high levels of missing data but does not improve. As we noted in the main text, this is likely due to how local degree correlations affect which nodes are added to the sampling frame during the process. Unlike the medium and high transitivity networks from the main text, the average degree of the sampling frame at the end of the $(\overline{G}, G)$ acquaintance immunization process are only slightly higher than the average degree of their whole network (Erdős-Renyi: going from 8.65 to 8.74, Barábasi-Albert going from 5.92 to 6.16, and both types of Salathé-Jones networks going from 10.00 to 10.02). While the mechanics of this process will be the subject of future work, the implications are promising and perhaps immediately useful to act upon, as our results suggest that an increase in the average degree of a sampling frame during the $(\overline{G}, G)$ acquaintance immunization process may serve as indicator as to how effective it will be.

References

1. Erdős P, Rényi A. On the evolution of random graphs. Publ Math Inst Hung Acad Sci. 1960;5(1):17–60.

2. Barabási AL, Albert R. Emergence of scaling in random networks. Science. 1999;286(5439):509–512.

3. Chen S, Lu X. An immunization strategy for hidden populations. Scientific Reports. 2017;7(1):3268.

4. Salathé M, Jones JH. Dynamics and control of diseases in networks with community structure. PLoS Computational Biology. 2010;6(4):e1000736.

5. Watts DJ, Strogatz SH. Collective dynamics of 'small-world' networks. Nature. 1998;393(6684):440.

6. Reichardt J, Bornholdt S. Statistical mechanics of community detection. Physical Review E. 2006;74(1):016110.

7. Hébert-Dufresne L, Allard A, Young JG, Dubé LJ. Global efficiency of local immunization on complex networks. Scientific Reports. 2013;3:2171.

8. Newman ME. Spread of epidemic disease on networks. Physical Review E. 2002;66(1):016128.

9. Hébert-Dufresne L, Patterson-Lomba O, Goerg GM, Althouse BM. Pathogen mutation modeled by competition between site and bond percolation. Physical Review Letters. 2013;110(10):108103.
