# Supplementary material for: Immunization strategies in networks with missing data
Source: PLoS Comput Biol. 2020 Jul 9;16(7):e1007897. doi: 10.1371/journal.pcbi.1007897 (PMC7386582; doi:10.1371/journal.pcbi.1007897)
Supplement: Attachment — Submitted filename: Response_Document_PCOMPBIOL-D-19-01722.pdf [file pcbi.1007897.s002.pdf]

Immunization Strategies in Networks with Missing Data (PCOMPBIOL-D-19-01722)

Answers to Comments

Samuel F. Rosenblatt[1, 2], Jeffrey A. Smith[3], G. Robin Gauthier[3], Laurent Hébert-Dufresne[1,2]

[1] Department of Computer Science, University of Vermont, Burlington, VT 05405
[2] Vermont Complex Systems Center, University of Vermont, Burlington, VT 05405
[3] Department of Sociology, University of Nebraska-Lincoln, Lincoln, NE 68588

March 6, 2020

We thank the reviewers for their time and for their thoughtful comments and suggestions. We have made substantial modifications to the manuscript and we believe that it is much improved. We have cut down the introduction and methods sections to make the document more readable, and have completely re-written the results section to highlight the major findings of the study. We have also added new analyses that include a wider range of network structures and input parameters to the epidemiological simulations. We address the reviewers' specific points below.

Answers to Comments from Reviewer 1

Point **1.1A**

This manuscript examines the spread of contagious elements in a network population. This is a simulation based study in which the authors examine eight different immunization strategies over a network with variable vaccination rate, transitivity, and information on the network itself. They then apply an epidemic model over many stochastic realizations to assess mean final epidemic size. They find tradeoffs, particularly in acquaintance immunization between information required and robustness to missing data. These results could be applicable to a number of epidemic settings.

Answer 1.1A— We thank the reviewer for their constructive assessment of our work.

Point **1.1B**

Between the main text and supplement, this is a very dense manuscript. This is somewhat the manuscript's weakness as there is a good amount of notation, while the manuscript strives to provide a general approach to contagion phenomena. However, there were multiple times in the manuscript where I was uncertain what exactly was being modeled and,

therefore, the key implications of the results. My main reservations are based on how the epidemic model was formulated over the network, and what approximate disease and population were being examined. Honing in on modeling a specific setting would strengthen the manuscript.

Answer 1.1B— We agree with the reviewer that the manuscript was too dense in our original submission. We have edited the manuscript to make it clearer and easier to read. We have substantially reduced the length of the introduction and streamlined the background and theory sections. We have also clarified important details in the methods section and made substantial changes to the results section.

We agree with the reviewer's concern that we were unclear about the conditions under which our results were applicable. Our intent was to examine the effect of missing data on immunization strategies for a generalized outbreak model, for better comparability with the existing literature on targeted immunization. We vary the inputs to our epidemiological simulations to show how the initial results are extended to a wide range of outcomes. In this way, our results are not based on a single case, eg. just HIV, but instead, we look at a range of cases where each case could be thought of as a different type of infection, with its own infectiousness and recovery rate. We look at these infection types in different network settings, discussing how different networks would allow different kinds of infections to spread. However, we agree that it would be useful to understand how each case we examine approximately relates to a particular disease. Thus we now include in each figure caption a simulation-based estimate of R_0 for every combination of network and infectiousness parameter we test.

Point **1.1C**

In section 2.4 and beyond, it was unclear to me what situation / disease was being modeled. There is a substantial discussion about HIV spread in section 2.1, and the networks utilized seem based on that, so I assumed the disease being modeled was HIV. However, a Susceptible-Infected-Recovered model was used. Disease-specific time units aren't mentioned here, but if one supposes they are in days the infectious period is 5 days which would indicate more of a childhood disease or a flu-type pathogen.

Answer 1.1C—This was unclear in the original description and we thank the reviewer for drawing our attention to this oversight. The heavy focus on HIV was meant simply to provide a concrete example of a situation where there was a hidden population, paired with a contagion that has other forms of intervention besides vaccination which can effectively immunize a node, such as rehabilitation, which would remove the node from the population of injection drug users. However, several of the methods of non-vaccination intervention we used as examples are not well approximated by complete immunization, and the lengthy discussion muddied our goal of a more general model of contagion. We now make clear that our results are meant to apply to a general range of cases, rather than a specific disease, although they map onto potentially realistic epidemic spread. As explained in response to point 1.1D, we now explore a wider range of potential outbreak size to showcase the generality of the model and results.

We use the SIR model as it is general and widely applicable to the scenarios we have in mind. Similarly, we investigate enough sets of parameters to apply our approach on all possible outcomes of the model: from minimal outbreaks that are contained by the intervention to large epidemics that span almost the entire population.

In the context of the studied interventions, the scenarios we consider are applicable more generally to potential disease epidemics that are more clearly modeled on networks and for which data is difficult to collect, such as those which spread through sexual contact or shared drug use, including HIV, HCV, and syphilis. For the high school friendship network (see additional analyses), the disease scenarios would correspond to infections like influenza, mononucleosis, or anything else passed through social contact networks of high school students.

Point **1.1D**

*The authors do utilize a range of parameters, but they are not so drastically di*fferent.

Answer 1.1D—

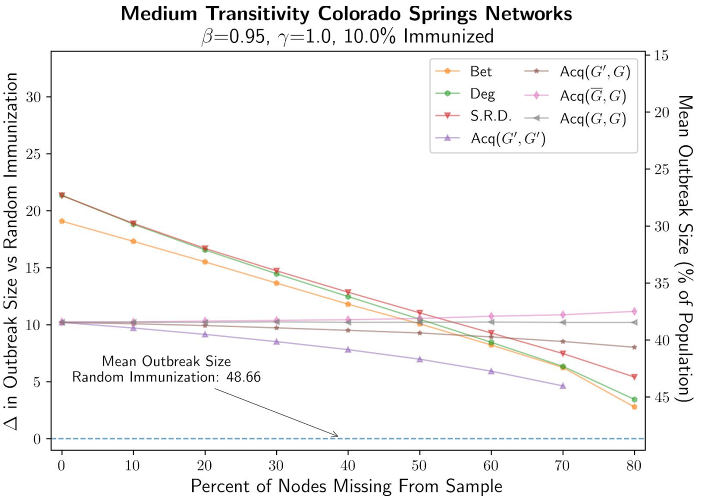

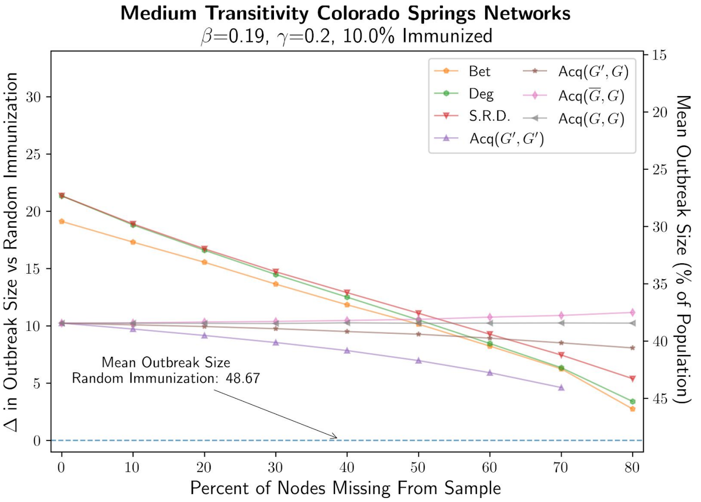

We agree. Initially, we chose our range for β and γ for comparability: such that the "middle values" for β and γ (0.08 and 0.2, respectively) were the same as those in one of the papers we cite the most we cited the most (Gong et al., 2013) and equal to one of the values used another of the papers we cite the most (Salathé and Jones, 2010). That did lead to a somewhat narrow range of epidemic sizes.

For our new range of parameters, instead of focusing on comparability, we used a new set of criteria to choose them. We chose our value for γ to be 1.0 so that the values for β would be easy to interpret relative to recovery rate. This is equivalent to setting time units to be equal to the expected time for recovery. Then, at the reviewer's suggestion, our next focus was creating a wider range. Our lowest value of β (0.2) was chosen to be just over the epidemic threshold with random immunization for all of the networks we test in the main text. To establish the rest of the range, we then increased β in increments of 0.25 until each of the four kinds of networks that we test in

the main text yielded outbreak sizes of over 50% of their population when random immunization was applied. With these values, the SIR dynamics spans almost the entire range of possible epidemic sizes.

Though displaying figures for all the values of β that we tested on would be impractical in the main text, we do include results from all the values of β for the four ensembles of networks we use in the main text in the supporting information (Subsection 2.3).

Point **1.1E**

Depending on this, it's unclear how appropriate the chosen base network is. For example, childhood diseases will require more information on classroom, household location and sibling, age, and school (see Hagelloch data and manuscripts from Groendyke & Welch). These networks will certainly be different given children vs adults mixing patterns.

Answer 1.E—In addition to adding a wide range of disease model parameters, we have also incorporated an additional network context into the analysis. This additional network is based on friendships between adolescents in high school. This allows us to explore the model in a context with potentially different structural features and infection risk. We thank the reviewer for their helpful suggestion.

Point **1.1F**

Without knowing how R0 was calculated, it was unclear how transmissible a pathogen was being discussed, which was then hard to reconcile with the vaccination levels of 2.5% to 10%. For some diseases / populations / eras this could be possible, but given something like a flu or measles, we may expect these to be much higher, up to 50% or even ≥ 90%.

Answer 1.1F— At the reviewer's suggestion, we have included a measured value of R0 for all results as well as a and a description of how it was calculated. The measured value of R0 for a given ensemble of networks for a given pair of SIR parameters β and γ is included in the figure caption of every plot. R0 is measured directly in the simulations by averaging the number of secondary infections caused by nodes reached early in the outbreak (we use the second infectious node to avoid the impact of saturation and of the choice of patient zero). This value was calculated numerically using the same python package that implements our SIR simulations (https://github.com/gstonge/spreading_CR#estimation-of-the-basic-reproduction-number-r0). For every network we test on, we use this package to set up a simulation with no immunization, and then play out just the beginning part of this simulation 10,000 times, measuring the number of cases that the second infected node causes. We hope that the reporting of this value with all results, along with a discussion of how to interpret β and γ in relative time units will make it clear how transmissible a particular contagion is on a given network. This turned out to be an excellent suggestion. The inclusion of R0 values for reference will certainly make this paper more relatable to a wider audience, and perhaps contribute to its role in policy.

Additionally, to address the reviewer's point about the low immunization levels, we scaled up the range of immunization levels we tested on from 2.5%, 5%, 7.5% and 10%, to our new range of 5%, 10%, and 15%, and we conducted a limited test of the much higher immunization level of 50%, which we show here for the reviewer, and include in the supporting information for others with the same question as the reviewer.

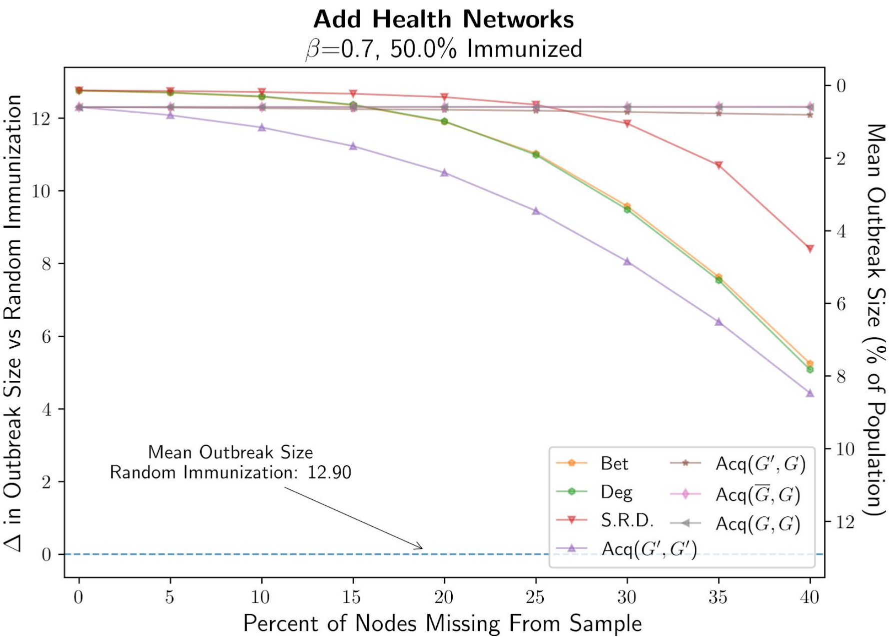

Observe that the x-axis of this figure extends only to 40%, instead of the range up to 80% missing data that we use in all other tests. This is by necessity when immunization levels are high. Immunization strategies that can only immunize nodes in the observed network are therefore by definition limited in their immunization coverage to, at maximum, the percent of the network data they have available (100 - Percent of Nodes Missing From Sample). However, at that maximum, the notion of "targeted" immunization is meaningless, as immunizing 50% of the network, when there is only 50% of the network available to immunize requires immunizing all nodes available, with no discernment based on strategy. Because the focus of this study was on missing data, we decided that exploring a wider range of missing data was more important than exploring a wider range of immunization coverage levels. Thus our initial highest level of immunization was 10%. We have since raised it to 15% at the reviewer's suggestion, but would not want to go much higher for our main results as it would mean reducing the highest level of missing data.

Point **1.1G**

I also wasn't sure if these vaccination levels were sustained throughout the epidemic duration.

Answer 1.G—We now make clear that consistent with the body of targeted immunization literature using SIR models, we consider a static population with a single contagion and a 100% effective immunization throughout the duration of the epidemic.

Point **1.1H**

Lastly, I assumed vaccinated nodes were moved to the 'Recovered' class of the model, but then is everyone assumed to be susceptible at the start of the epidemic?

Answer 1.1H—We now make clear that everyone is assumed to be susceptible before the immunization is "administered" and the vaccinated nodes are moved to the recovered state.

Point **1.1I**

Overall in this section I had trouble following what specific pathogen / population was being modeled and it was thus hard for me to interpret the results of epidemic final size.

Answer 1.1I— We now make clear that our model and approach are meant to be as general as possible, while still giving specific examples of what pathogens might be spreading on the different considered datasets. This should make it easier to interpret the results. In addition, with multiple scenarios, it is easier to see how the final epidemic size changes with the parameters of the epidemiological simulations. To help intuition for readers familiar with disease modeling, we now also provide the R0 for all of our simulations. In interpreting our results, we then focus more on the rank ordering of the strategies rather than on the final epidemic size. We examine the relationship between missing data and the efficacy of the strategies, noting cross over points where one strategy overtakes another as the optimal choice. The final epidemic size and all disease parameters are, however, available for the reader to view on the plots and captions provided in the main text and supporting analyses.

Point **1.2**

I understand plotting constraints given the many scenarios, but it seems necessary to add some measure of variability to these final size estimates given the stochastic nature of network epidemic models. From a practical point of view, this is important as if a particular strategy leads to a particularly long distribution tail, e.g., "super-spreaders" a la Lloyd-Smith, 2005, that may be an undesirable result for those planning an intervention strategy. A relatively simple summary static such as fitting negative binomial distributions to the epidemic final sizes would be beneficial as a result. The caption for Figure 2 does mention that 95% CIs were smaller than the marker size which, given the stochastic epidemic model on a network, seems slightly unusual

Answer 1.2.—

The main plots capture the mean epidemic size for each strategy over all simulations, calculated by taking the grand mean of the distribution of 5000 outbreak size means, where each of those is the mean size of 2000 outbreak simulations on one of 5000 networks. The distribution of mean values itself has little variability given the large number of simulations we employed. We agree, however, that a researcher would like to have a sense of the shape of the outbreak size distributions of each strategy for individual simulations; especially given that the underlying distribution is often bimodal, consisting of epidemics that stochastically die out or invade the network. Instead, we simply display the pooled distributions of the individual simulations directly with a series of histograms in (figures S8 - S15, with a description in the caption of S8), which we refer to in the main text in the context of a discussion about the distribution shape and variability from lines 387 to 398. We keep our global metric as the mean epidemic size, rather than the mean size for epidemics over a certain threshold (as is common), because we think simple averages are easy to interpret and because thresholding methods such as those employed in Salathé and Jones 2010 yield values which do not have a consistent meaning when comparing between a bimodal distribution (where the threshold is clear) and outbreak distributions near the epidemic threshold. This is exemplified in figure S11, when comparing the outbreak distributions of the degree strategy at 0% missing data to the degree strategy at 40% or 80% missing data. Note that comparing these kinds of distributions is essential to our study due to the wide range of parameter values we test on.

Point **1.3** *The problem of missing data seems two-fold. There is the case where information on particular nodes in the network are missing, which is discussed here. But there is also the case of missing epidemic information. This may be incorporated into missing node information itself, but it seems there could be a very likely scenario where you have information on node X in terms of their contacts and connectivity, etc. However, in a real world setting you may not necessarily know if X gets infected and then spreads. Could this be incorporated into this model? I'm not necessarily suggesting to do that here, as there are many results discussed, but a discussion, and more clear description of the assumptions (e.g., 100% reporting), of this topic in the discussion section may help target the manuscript to the type of epidemic scenarios for which these results would be most applicable.*

Answer 1.3— As discussed further in point 1.9, we now make clear that immunization is a preventative, rather than a responsive intervention. This means that all immunization is done prior to first infection, and our network

measures attempt to measure risk in the contact network, not in an infection network. Knowledge of which nodes are infected is not incorporated into any strategy.

Point **1.4**

Line 23: Unclear phrasing at the start of the sentence: pathogens are not inherently negative.

Answer 1.4—The sentence has been rephrased. It now reads: "If negative contagious pathogens or content spread over network connections, what kinds of interventions will be most effective at inhibiting diffusion and outbreak size."

Point **1.5**

Line 33: Immunization maybe should be changed to intervention if reduction of contact is included

Answer 1.5— We now make clear that we model a procedure that irreversibly prevents infection for a particular node with 100% effectiveness. We have also condensed the discussion and references to interventions that should not be approximated as immunizations. Additionally, we have included as a limited analysis in the Supporting Information (S24), what some of our results would look like if we did not make the assumption of a 100% effective immunization.

Point **1.6**

Line 216: Unclear what 'attempted to saturate the population' means

Answer 1.6— The sentence has been rephrased. It now reads: "Researchers attempted to identify the entire at-risk population in the city and include them in their study."

Point **1.7**

Line 231-238: It would be helpful to give a real world example of what a low transitivity (0.01) network would represent

Answer 1.7— This low level of transitivity could be found in a predominately heterosexual sex network. If male a has sexual relations with female b and female c, there is only a small probability of female b and female c having sexual contact between themselves.

Point **1.8**

Line 246: Does clustering mean the same as transitivity here? If so I would use consistent language

Answer 1.8— We have updated our language so that transitivity is consistently used.

Point **1.9**

Line 383: How was vaccination implemented into the SIR model? Is there vaccination during the course of the epidemic? If so, is it targeted? If not, it may be worthwhile to mention that in the discussion section as a limitation.

Answer 1.9— We thank the reviewer for pointing out some shortcomings of our methods section. Indeed, vaccination during an ongoing epidemic may be the scenario that a reader is more familiar with, but our goal in this paper was to point out the importance of one of many assumptions in the targeted immunization literature (which assumes all immunization is done prior to first infection), and to introduce a general model to demonstrate how removing the assumption of perfect data changes some of the intuitions that have coalesced in this literature. However, we believe that the findings from the targeted immunization literature still have much to offer. So instead of simply designing our model to incorporate as much realism as possible, and thereby making it difficult to draw comparisons to the existing literature, we see this study as the

first step among several, that slowly take the highly abstracted problem of targeted immunization and apply additional realism modularly in order to connect the abstracted version of the problem to a more practical policy suggestion. The reviewer's suggestion is a good one, in that they point out what is one of the larger differences between targeted immunization as an abstract problem and how the average person thinks about immunization. As such, we have added this to the discussion as one of the extensions of this model that would like to see taken next.

Additionally, the reviewer's question alerted us to an oversight we made in the language of the paper. In the first submission, we had used the terms "removed" and "immunized" interchangeably. This was because in an earlier version of the computational model for this study, the way that the vaccination was implemented in the code was by removing the targeted nodes from the data structure representing the network before the outbreak began (which is mathematically equivalent to moving them to the 'recovered' state as long as this is accounted for in the calculations involving network size and such) . However, the model used currently, and which was used to generate the results for the initial submission, implements immunization by initializing the network of the spreading-process object with the targeted nodes in the 'recovered' state before the process begins. The example given here in the documentation of the SIR simulation package we use demonstrates how this works.
https://github.com/gstonge/spreading_CR#initialize-the-network-with-recovered-immuned-nodes Additionally, while the extensive amount of new analysis carried out during this revision process prevented us from being able to keep our code clean enough to provide with this revision, cleaning and documenting our code is a priority post-revision submission (and would certainly be done before publication if accepted) and so future readers of this paper curious about the actual implementation of immunization computationally would be able to do investigate easily by opening the documented code.

Point **1.10**

Line 383: The actual model uses immunization as vaccination, but the language in the introduction states that immunization is any intervention. This language should be standardized throughout the manuscript

Answer 1.10— We have now made clear in the text that the kind of targeted intervention we are modeling is based on immunization. We also now consistently discuss immunization levels rather than vaccination levels. The term 'immunization' here refers to anything that irreversibly breaks the contagion-transmitting pathways for a particular actor for a particular 'contagion'. In practice, this could refer to a vaccination, but other interventions could also conceivably permanently limit the contagion's spread. Throughout this paper, we assume that the intervention provides immunity through vaccination.

Point **1.11**

Line 395-399: What are the time units here? What is the R0 of the pathogen, and how is it calculated?

Answer 1.11—

Since we are only concerned with the final size of an outbreak, the unit of time in the model itself is arbitrary and can mean days, weeks, months, or anything in between. We now fix the recovery rate to one, which means that time is counted in "number of recovery periods". To help readers not too familiar with normalized time units understand the level of infectiousness used in different scenarios, we also include R0 as a summary measure in the analyses. As mentioned earlier, R0 is measured directly in the simulations by averaging the number of secondary infections caused by nodes reached early in the outbreak (we use the second infectious node toi avoid the impact of saturation and of the choice of patient zero).

Point **1.12**

Description of the SIR model: when does the epidemic end?

Answer 1.12— The exclusion of this detail was an oversight. We thank the reviewer for pointing it out.
We have added these details to the text. "The SIR model runs until there are no longer any infected nodes. We assume the infection runs its course without further intervention."

Point **1.13**

Figure 2 caption: What does it mean for a strategy to not be able to converge to an immunization level?

Answer 1.13— We agree that further clarification was needed. The stochastic strategies relied on being able to eventually identify enough nodes to meet the desired immunization level. However, in very rare scenarios, this was not possible, as high levels of missing data yielded networks where the randomly selected nodes had few (or no) neighbors. In such cases the stochastic strategies failed to identify the specified level of nodes to immunize. This clarification was added in the caption of figure 2.

Point **1.14**

Point 1.14 Figure 2: What was the epidemic size in the absence of any intervention strategy? If it was for example 90% of the population, then the random intervention may be still somewhat impressive. But again this depends on what disease is being modeling here.

Answer 1.14— We now include the percent infected with no immunization for comparison in the figure captions of figures 2, 3 and 4.

Point **1.15**

Final paragraph for the discussion: will code be made available to adapt this analysis to other situations, networks, and models?

Answer 1.15— Yes. The code will be made available. The simulation code is already available (see previous URL for the github repo). To help future researchers be able to adapt the code to other situations, networks and models, we are currently developing the full software and documentation, and will make it public prior to publication.

Answers to Comments from Reviewer 2

General Assessment *This paper examines the optimal way of allocating vaccinations among a population when the transmission network over which the disease spreads is known, but with error. A large body of previous work over the past 20+ years has focused on optimal vaccination strategies when resources are limited, but has always assumed that everything about the network is known. In parallel, a large body of work from network sciences has focused on estimating network properties in an unbiased way when there is missing data and different biases in sampling strategies. This paper combines these two ideas to understand what network-based vaccine distribution schemes are optimal when networks are imperfectly sampled.*

Overall I think this is a really nice addition to the field. The authors clearly show that some network-based vaccination strategies are still way better than random allocation strategies even under realistically high levels of missing data. They show how the optimal strategy depends on some properties of the network, the nature of the missing data, and the details of how the network is used to allocate the vaccine. Their methods are easy to follow and the introduction is a very thorough review of the field. This paper is an important step in connecting

the mainly theoretical work done on vaccine allocation with the real-world constraints of mapping out risk networks for real diseases.

We thank the reviewer for their positive and very encouraging assessment of our work.

Point 2.1

Some parts of the paper are really wordy and it takes a really long time to get through. This includes the introduction (Section 1) and the Materials & Methods (Section 2). And the Results section reads more like a Figure caption than an appealing narrative of some very nice findings! I think that all of this could be a lot more concise and reader-friendly with some careful editing.

Answer 2.1—We agree with the reviewer that the initial document was too long and difficult to follow. We thank the reviewer for their suggestions for editing the manuscript. We have substantially reduced the length of the introduction and material and methods section. The results section has been reorganized and largely re-written. It is now focused on the findings, making the results clearer and easier to follow.

Point 2.2

The results section of the paper is pretty small compared to the lead up. I don't think the conclusions are adequately explored for their relevance in slightly different scenarios. I think that the authors could easily explore a few more scenarios to fill out this section to the size normally expected in a PLoS Computational Biology paper. For example, why not explore some other network structures that are very different from the Colorado Springs network? Why not consider some other infection models, like SIS? These are mentioned in the Discussion as future work but would be more appropriately included in this manuscript

Answer 2.2—We agree with the reviewer that the initial results shown in the main text were too narrow. We have thus added completely new analyses to the paper. These analyses generalize the results and allow us to explore the robustness of our findings. The results section now includes an extended discussion across a wider range of infectiousness types. We also include an additional network context of high school friendships with completely different features then the three Colorado Springs networks already discussed in the text. Overall, our results include variation along a number of dimensions, including: network type, infectiousness, immunization levels, and missing data levels. We think overall this is a sufficient amount of variation to demonstrate the generalizability and robustness of our results. Moreover, we have kept the simulations on random networks in the supporting information and have also added limited analyses reproducing our main results under imperfect immunization as well as larger immunization coverage (Figures S22, S23, and S24).

Note that we still focus on SIR dynamics because we are concerned with short-term epidemic dynamics and point outbreak. In long-term SIS-like behaviour, the available data and intervention would both co-evolve along with the disease. SIS models are thus outside the scope of this paper, but could be an interesting avenue for future work.

Point 2.3

The Author Summary is just as technical as the Abstract - not appropriate for a lay scientific audience

Answer 2.3—We have now re-written the Author Summary to make it more accessible to a lay scientific audience

Point 2.4

Line 25 - Papers from year 2000 would not be characterized as "recent work" by most people

Answer 2.4—We've changed "recent work" to "prior work".

Point 2.5 *Most of these citations are to extremely theoretical papers by physicists. Do you have any examples of people in the field of infectious disease epidemiology considering network-based immunization strategies? Can you convince the reader that there any real world practicality to such ideas? I'm not sure just citing papers of immunizing healthcare workers counts. This is an entire group of people, and doesn't really use any specific concept of networks.*

Answer 2.5— The theoretical literature has consistently suggested for decades that targeted immunization is effective (emphasized in the text by including an abundance of citations in the first paragraph of the introduction), and non-immunization network-based interventions have been found to be effective in practice (examples added at the end of the first paragraph in the introduction). However, despite calls within the field of infectious disease epidemiology to act on these ideas (https://bmcinfectdis.biomedcentral.com/articles/10.1186/s12879-018-3093-x), so far, we are unaware of any applied intervention campaigns in the field of infectious disease which utilize directly the strategies described in the targeted immunization literature. Though the reasons for this are not entirely clear, we believe one of the main factors is precisely what the reviewer is alluding to: the organizers of interventions view the current body of targeted immunization literature as too theoretical and its assumptions too far afield from the reality of their individual cases that it is not relevant to them. Our goal in this paper is to begin to reconcile this issue by removing one of the most unrealistic assumptions of previous targeted immunization literature. Hopefully, showing that the practice of targeted immunization is still extremely effective in a slightly more realistic scenario will suggest to expert readers that targeted immunization may be effective in the real world.

Point 2.6 *Overall this section[Materials and Methods] is very wordy. Could be shorted by just writing more concisely, and with some details moved to figure captions*

Answer 2.6—We have reduced the Materials and Methods section, moved some details to figure captions, and reduced the number of subsections by moving the important parts of what was subsection 2.5 into 2.4. The section is now easier to read and more focused. We do however feel like we need to completely describe the strategies and simulation methods before discussing the results.

Point 2.7A

Figure 1. Caption needs more details. Does a degree here correspond to either a close friendship, sexual contact, or drug co-use, or just one of these relationships?

Answer 2.7A—We discuss the tie content in the main text, and have added a description in Section 2.1. The text now makes clear that the ties are an amalgamation of friendship, sexual contact and drug use.

Point 2.7B *[Figure 1] Also there is a big enough population size that it would be clearer to have each degree as its own bin instead of two degrees per bin, so it would be clear how many individuals have zero vs one vs two degree, etc.*

Answer 2.7B—This is an excellent suggestion. We have now updated the figure to show more differentiation in terms of degree.

Point 2.7C *It would also be nice to show an image of the graph structure. Degree distribution doesn't fully capture the structure of a network, like how much clustering there is.*

Answer 2.7C—We thank the reviewer for this suggestion. We now include visualizations of the networks used in the simulations in Figure 1.

Point 2.8 *It would be also worth mentioning re the Colorado Springs network that HIV is only one of the many infections that is commonly spread by persons with infection drug use; Hep A, B, and C, as well as other STDs, are also common in these populations*

Answer 2.8—We now make clear through the text that this model is intended to be general. We have removed specific references to HIV, and we have made clear that the infection of interest could refer to a wide range of infections including Hepatitis, syphilis etc.

Point 2.9

And PWID stands for "PERSONS who inject drugs".

Answer 2.9—We have now fixed it in the text.

Point 2.10 *Line 292 - Degree immunization - It's not totally clear what you mean when you say "selects nodes based on network degree". Do you rank based on network degree then choose the top X? What if there are ties? Or is selection random but proportional to degree? Directly proportional to degree or on a log or other scale? Same question for some of the other strategies*

Answer 2.10—For Degree immunization, Betweenness immunization, and Self-Reported Degree immunization, we rank based on the corresponding measure and then choose the top X, breaking ties randomly. We describe the process in the methods section.

Point 2.11

Section 2.3: Are there ever scenarios in which edges are missing at random, even though the nodes they may be connecting are in the network?

Answer 2.11—This is a realistic scenario that will hopefully be included in future research. We discuss the possibilities of exploring different types of missing data in the conclusion.

Point 2.12 *Do you also consider immunization under FCD and RDS, as the previous papers you cited around line 170 did? Just to repeat their results and compare to yours to be as comprehensive as possible?*

Answer 2.12— For this manuscript we limited our analysis specifically to incomplete sampling specifically in the form of missing nodes at random. We have already begun a project looking at the effect of non-random missing data in terms of the Boundary Specification Problem, and in the future, a comparative review of the effect of different kinds of missing data on targeted immunization would be a good place for a more systematic replication of the results these studies and any that come in the near future. However, given the wide range of scenarios we did consider, this unfortunately is out of the scope of this paper.

Point 2.13 *Section 2.4: I'm a bit confused by the description of the SIR model. First, you say individuals "pass the infection to all of their nearest neighbors in the network". However, that is not a correct way to simulate a stochastic infection model. Each infected node should have a constant probability (gamma) of recovery per unit time, and each susceptible node should have a probability dependent on its number of infected neighbors* (*beta x* $n_{infected}$) *of becoming infected. The exponentiated equations the authors write involving time period Delta only hold if time Delta is very small, such that the probability that more than one event happening in the same time interval is negligible (e.g. there is no chance that an infected neighbor will recover before it infects you).*

Answer 2.13—Everything the reviewer wrote is correct and the original sentence was indeed confusing. Transmission is possible (so the sentence shouldn't be definitive) and occurs individually and independently for every contact with susceptibles around a given infectious individual (so the sentence shouldn't say "to all"). Moreover, since our simulation code uses continuous time, we should not describe the SIR process as discrete. All the details of our SIR simulations are given in the following reference: St-Onge G, Young JG, Hébert-Dufresne L, Dubé LJ. Efficient sampling of spreading processes on complex networks using a composition and rejection algorithm. Computer Physics Communications. 2019;240:30–37. The description of the SIR model was changed to: "Individual SIR simulations assume that there is an initial infected seed that can potentially pass the infection to their nearest neighbors in the network; the infected neighbors could then pass the infection to their own nearest neighbors, and so on, with nodes recovering out of their infected state after a stochastic amount of time related to a fixed recovery rate value. More precisely, every infected node infect their susceptible neighbours at rate β and recover at rate γ. The SIR model runs until there are no longer any infected nodes"

Point 2.14 *This section[Results] reads like a figure caption. It would be much clearer to describe the FINDINGS, not narrate the graph- ics (e.g. what is on the x vs y axes)*

Answer 2.14

We have thoroughly re-organized and re-written the results section. The results section now focuses on the findings, making it easier to follow.

Point 2.15

Figure 2 caption: Include explanation of abbreviations in legend, e.g. S.R.D = Self-reported degree

Answer 2.15—We now include more informative labelling of our legends in our captions for figures 2, 3, and 4, as well as keep the color associated with a particular immunization strategy constant throughout, even for different types of plot, such as the distribution plots S8-S15.

Point 2.16 *Figure 2/3/4 all look really similar. Is there not a more creative way to show some of these trends? For example, if you want to show the effect of changing transitivity, make a graph with transitivity as the x axis. Same could be said for % immunized. I personally would find it more intuitive if the y axis on all the graphs was not final infection size but the opposite, e.g. % not infected, so that higher values would be "better". It seems like the main trend of interest to someone designing a vaccination strategy is % reduction in infection vs %*

immunized, and how this trend depends on details of missing data, immunization strategy, and network structure, so it might be more intuitive to present the data this way.

Answer 2.16— More than highlighting the effect of changing transitivity and % immunized, we wanted to highlight that results of varying the level of missing data were qualitatively similar across a broad range of scenarios, which is accomplished by showing the main type of plot at extreme values of parameter space. However, in the case of changing the value of β, we did find an interesting trend there, and so in that case we do follow the reviewer's advice and create plots with β on the x axis (Figure 5). Additionally, we follow the reviewer's advice and redesigned the visualizations so that the y-values are something like the "opposite" of final outbreak size. We now show them as a difference in outbreak size versus random immunization, which can be thought of as "% saved by implementing targeted immunization instead of random". This highlights the differences across strategies and puts more effective strategies visually at the top of the figure. It also comes with the added benefit of reducing the spread of values on the y-axis between plots with different parameters, which allows us to fix the y axis between subfigures where doing so on the y-axis used previously would have made the results unreadable (for example, if the y-axis was still final outbreak size, and we fixed the y-axis for subfigures 4.i, 4.ii, and 4.iii, then all the lines in subfigure 4.i would be visually on top of each other. We have included this example below). For additional understanding and ability to compare these results to other papers which use % infected on the y axis, we kept that original axis as a secondary axis on the right of figures 2, 3, and 4.

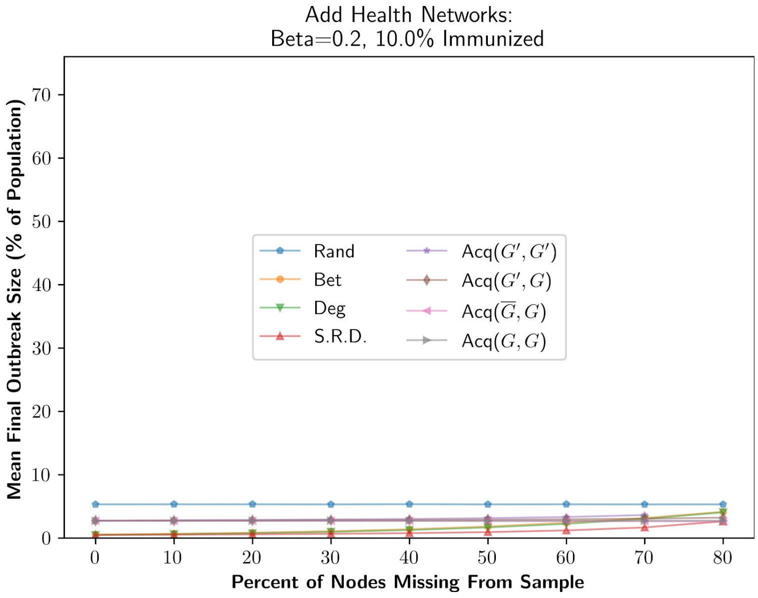

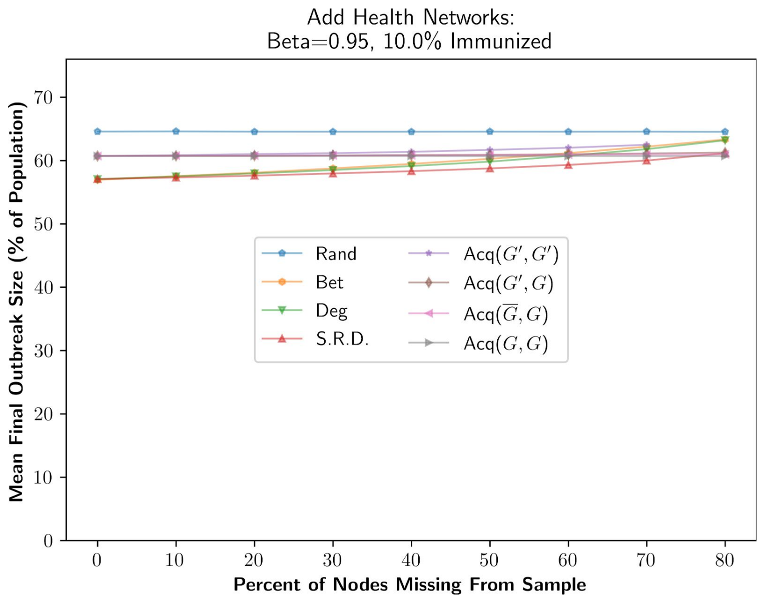

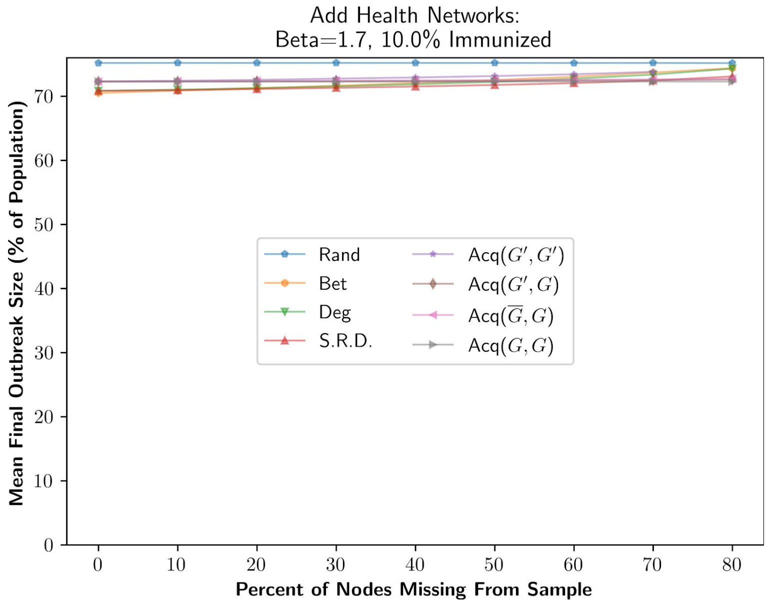

Point **2.17**

I think there are many times when you cite Figure 3 but you really mean to cite Figure 2.

Answer 2.17— We have fixed this error.

Point **2.18**

I found the results section rambling and hard to follow. Instead of starting each paragraph with "Figure X shows . . . ", start with a topic sentence summarizing a finding you observed, e.g. "Random immunization was the worst strategy at every level of immunization coverage and missing data". If the finding is seen in a particular figure, add it as a reference, e.g. "(Fig X)".

Answer 2.18—We thank the reviewer for their very approachable advice on how to revise the results section. We have thoroughly re-written the section to be much more concise and have a much clearer thread. We now no longer point out many details about specific plots, but instead highlight the most salient parts of the results and leave the reader to draw their own conclusions about specifics.

Point 2.19

Line 609: I don't think you have defined yet what you mean by robust vs efficacious.

Answer 2.19—We now define efficacy and robustness in section 1.2
